# The dynamic nature of cereal food webs challenges the suitability of snapshot sampling for assessing ecosystem services

**DOI:** 10.1038/s41598-025-25603-2

**Published:** 2025-11-24

**Authors:** Pedro Nuno Branco Leote, Michael Traugott, Oskar Ragnar Rennstam Rubbmark

**Affiliations:** Applied Animal Ecology Research Unit, Department of Zoology, University of Innsbruck, Technikerstrasse 25, Innsbruck, 6020 Austria

**Keywords:** Time series food webs, Network complexity, Community turnover, Invertebrate food webs, Cereal crops, Biological control, Agroecology, Ecological networks, Ecosystem services

## Abstract

Current theory of food web dynamics is based on mid- to long-term data. However, the short-term dynamics are still poorly understood, as the technology to study them is not readily available. We addressed this gap, by sampling invertebrate food webs bi-weekly, with generalist and specialist predators, herbivores and detritivores as prey, in barley fields over two years. We measured weighted connectance, community composition and link rewiring dissimilarity, as well as species-level specialisation of predators. We expected the connectance to be lowest, and both dissimilarities to be highest during the mid-season, whereas specialisation was predicted to follow prey abundance. We found differing trends across years, with connectance declining over time, and community and rewiring dissimilarity fluctuating more in the second year than the first, when an aphid infestation occurred. Specialisation varied across the season, with aphids and cereal leaf beetles being consumed by more predator species as their abundances peaked, and by fewer before and after that, while springtails were universally consumed. Our results show that agro-ecosystems are highly dynamic, and that food webs undergo continuous restructuring even over very short timeframes; potentially leading to a considerable systematic under- or overestimation of ecosystem functioning assessment.

**Supplementary Information:**

The online version contains supplementary material available at 10.1038/s41598-025-25603-2.

## Introduction

As seasons progress and habitats change, it is well known that there will be phenological shifts in the communities and interaction networks, formed by species that inhabit most temperate ecosystems^1,2^. Furthermore, we also know that such changes, whether natural (e.g. seasonal variation, phenology, weather) or human-induced *(e.g.* agricultural management such as the application of chemicals, tilling, harvesting), can influence the strength and reliability of ecosystem services that human food production relies on^3^. However, to understand how to promote ecosystem services, we first need to understand the changes that the species communities that inhabit agro-ecosystems go through. Not least, because we know from theory that timing, functional roles and redundancy will be decided by a community-wide process, that depend on seasonal changes that occur within a habitat^4,5^.

Arable systems are a sub-type of agro-ecosystems, where the soil is usually ploughed, and habitat changes over the course of a year are quite apparent. In just a few months, non-perennial crops, such as cereals, undergo a cycle of sowing, growth, ripening and harvest, each of which is associated with a starkly contrasting habitat setting^6^. However, so far, we have a relatively poor understanding of what these changes mean for the food web interactions that live within arable fields. It is, for example, likely that food webs, in response to habitat changes occurring during the crop growth, will go through cycles of greater complexity when resources are abundant and lesser complexity when resources are scarce^7^. Food web changes also occur because consumers will modify their behaviour, by adapting their realized niches to either avoid competition or explore new resources as different food sources become more or less available^8^, or in response to phenological changes in the predator community^9^. However, hardly any empirical data is available on how food webs respond to changes in the ecosystem induced by phenology and resource availability.

There are several reasons for why data with sufficient temporal resolution is missing and has been difficult to attain, such as the logistics required for the repeated sampling, the methodological challenges of developing assays and/or primers with the necessary balance of specificity and sensitivity, the costs of infrastructure and equipment needed, or the knowledge to curate and analyse such data^10–12^.

Here, we address this important data and knowledge gap by using a molecular analysis of trophic interactions between generalist (ground and rove beetles, and spiders) and specialist predators (lacewings, ladybugs and hoverfly larvae), detritivore (earthworms and springtails) and herbivore prey (aphids and cereal leaf beetle). This work is a follow-up to two previous articles, using the same community and diet data, the first focusing on the effects of fertilisation on prey abundance, intraguild predation and biological control^13^, and the second focusing on fertilisation, network-level specialisation and diversity^14^. Both are a part of a larger study we conducted in Central European barley fields, where we investigated food web interactions, as well as changes in communities, every two weeks throughout the cereal growth period, in replicated fields across two years. The fields were also fertilised on half of their respective areas, to induce different baseline primary productivity and prey abundances^13,15^. Our system is characterized by herbivorous insects, including the cereal leaf beetle *Oulema melanopus*, and three species of aphids that commonly occur in cereals, *Rhopalosiphum padi*, *Metopolophium dirhodum*, and *Sitobion avenae*^16,17^. Each of these species is attacked by a suite of natural enemies, among them generalist predators^18–21^. These are thought to reduce the chance of pests to become established in fields, whereas once they have reached higher densities, they are more effectively controlled by specialised enemies that are drawn to highly infested fields^22^. Outside of these periods of greater prey abundance, detritivore prey such as earthworms and springtails are valuable food sources, that can help sustain predator populations outside of the cereal growth season^23^; affecting adult populations, and their potential for pest control, in the following crop season^24^.

We devised three hypotheses, which conceptualize how food webs change according to our current understanding of food web and functional agro-ecology. First, the increase in detritivore and herbivore prey abundance on the fertilised side of the field and the lower competition (observed in^13^, should allow predators to explore a wider dietary niche early in the season, which should be reflected in a greater number of realized trophic links. As such, we predict food web complexity, measured with weighted connectance^25^, to be higher in the fertilised treatment. Moreover, because connectance decreases with species richness^26,27^, we expect weighted connectance to be lowest during the middle of the cereal growth season, when primary productivity and diversity in the crop fields is expected to be highest^7,13,28,29^. In our second hypothesis, we expect that specialisation on individual prey *taxa*, measured with Blüthgen’s *d’*^30^, should change over time in response to the availability of each prey, and be lower in the fertilised treatment; where we expect that an increased prey availability should allow predators to have more overlapping diets. However, over time we also expect specialisation should increase, as lowering competition and interference also will enable predators to focus on preferred prey. Lastly, in our third hypothesis, we expect trophic link rewiring or community composition dissimilarity^31^ to reach a peak towards the middle of the season, in response to the drastic habitat changes that occur as the cereal ripens and many herbivorous prey species migrate away from fields.

## Results

### Food webs

**Fig. 1:**
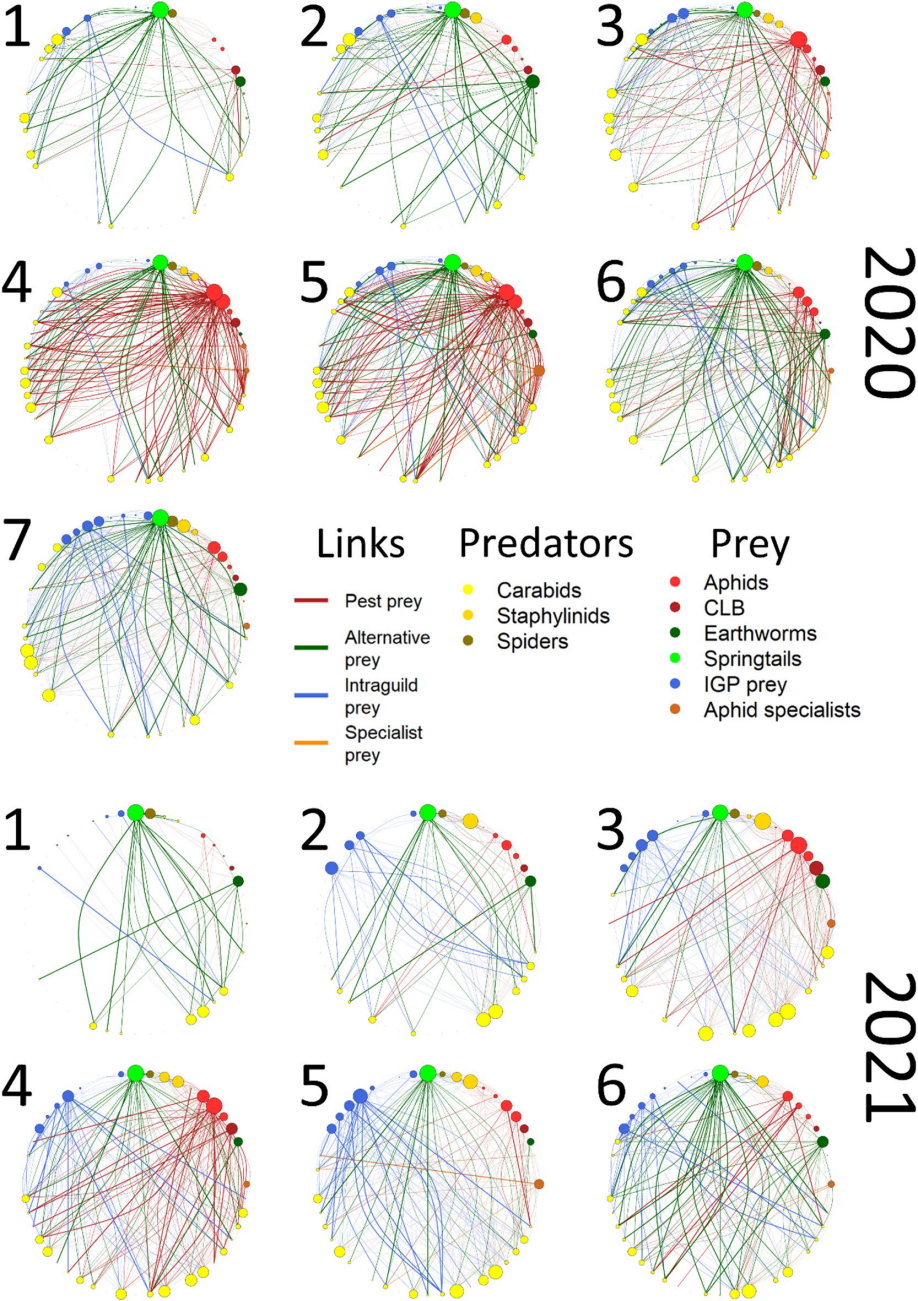
Food web diagrams for each sampling session (numbers) in 2020 and 2021, respectively, each group has been colour-coded, with node diameter corresponding to the eigenvector centrality and line width representing the diet detection proportion of the trophic link. From top to bottom in the figure; Predators: yellow – Carabid beetles, orange – Staphylinid beetles, brown – spiders; Prey: bright red – aphids, dark red – cereal leaf beetle, dark green – earthworms, bright green – springtails, bright blue – intraguild predation prey (beetles and spiders), dark orange – aphid specialists (hoverflies, ladybugs and lacewings).

The bipartite food webs for each sampling session of each year are described below (Fig. 1, but please see the supplementary Figs. 1 through 13 for each food web with higher resolution), to show the relative importance of each group (colour codedor), through the eigenvector centrality (node diameter), and diet detection proportion (line width). First and foremost, the number of trophic links established changed over time (supplementary Tables 9 and 10, and supplementary Fig. 14 for the link densities, and supplementary Tables 11 and 12 for eigenvector centralities). In 2020 and 2021, mean link density was 2.30 and 1.50, respectively, at the start of the season; then in the first year it reached a peak of 4.00 during the 5th session, while in the second year it was 3.44 during the 4th session. Finally, by the end of sampling, link density was 3.30 for 2020, and 2.87 for 2021.

Springtails (bright green) remained central throughout the season, with an eigenvector centrality ranging between 0.931 and 1 in 2020, and 0.950 and 1 in 2021. Earthworms (dark green), although less central than springtails, had a centrality between 0.613 and 0.826 early on and 0.628–0.800 late in the season in 2020, decreasing to 0.208 when aphids were at their peak in the 4th sampling session. In 2021, however, earthworms were more consistently central to the food webs, with a centrality between 0.646 and 0.870 with the exception of the 5th sampling session, when their centrality decreased to 0.422.

On the other hand, aphids (bright red) gained relevance in the middle sampling sessions in both years, rising in centrality from a mean of 0.121 and 0.131, to 0.571 and 0.598 in 2020 and 2021, respectively. The cereal leaf beetle (CLB, dark red), showed a peak in centrality of 0.573 and 0.858, during the 3rd session in both years.

The intraguild prey (IGP, bright blue), consisting of beetles and spiders, were more central at the end of the season in 2020, with a centrality of 0.395, when compared to the rest of sampling that ranged from 0.153 to 0.238. In the following year, centrality had two peaks, one in the 3rd session with 0.351 and later in the 5th session with 0.416. The specialist aphid predators (dark orange), encompassing ladybugs, lacewings and hoverflies (in larval stage), were relatively peripheral to the food webs, with centrality values as low as 0.034 in 2020 and 0 in 2021. Nonetheless, they displayed a peak of 0.289 and 0.200 centrality, during the 5th sampling session in the corresponding years.

Along with these descriptive metrics, several indices were calculated and described further below, with the results of the linear models testing the significance of sampling session and fertilisation treatment effects being summarized in Table 1.

**Table 1:** Summary of linear model statistical testing of the effects of sampling session and fertilisation treatment on weighted connectance, species-level specialisation, rewiring dissimilarity and community dissimilarity.

Metric	Year	Sampling session	Fertilisation
Weighted connectance	2020	*p* < 0.001**	*p* = 0.292
	2021	*p* < 0.001**	*p* = 0.869
Specialisation aphids	2020	Interaction, *p* = 0.025*	
	2021	*p* = 0.385	*p* = 0.014*
Specialisation CLB	2020	*p* < 0.001**	*p* = 0.249
	2021	*p* = 0.048*	*p* = 0.013*
Specialisation earthwm.	2020	*p* = 0.922	*p* = 0.346
	2021	*p* = 0.522	*p* = 0.359
Specialisation springtl.	2020	Not estimated	Not estimated
	2021	Not estimated	Not estimated
Specialisation beetles	2020	*p* = 0.272	*p* = 0.333
	2021	*p* = 0.012*	*p* = 0.740
Specialisation spiders	2020	*p* = 0.099	*p* = 0.589
	2021	*p* = 0.895	*p* = 0.634
Specialisation special.	2020	*p* = 0.071	*p* = 0.266
	2021	*p* = 0.597	*p* = 0.951
Rewiring dissimilarity	2020	*p* < 0.001**	*p* = 0.655
	2021	*p* < 0.001**	*p* = 0.934
Community dissimilarity	2020	*p* < 0.001**	*p* = 0.662
	2021	*p* < 0.001**	*p* = 0.884

*significant results, **strongly significant results. As mentioned in the *Specialisation* subsection of the Results, the linear models for specialisation on springtails could not be estimated, due to the majority of the data points (95% in 2020 and 97% in 2021) being zero.

### Food web complexity

**Fig. 2:**
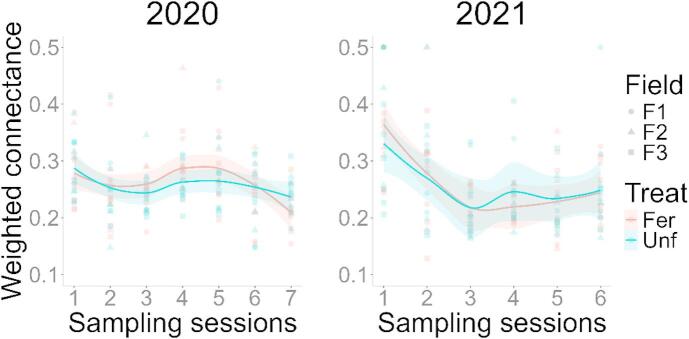
Food web complexity across sampling sessions and fertilisation treatments for 2020 and 2021, respectively. Complexity was measured by calculating weighted connectance^25^, using the *networklevel* function of the *bipartite* R package and the mean proportion of diet detections as weights. The lines correspond to loess smoothing, using the *geom_smooth* function of the *ggplot2* R package, with the standard error as the shaded areas.

The food web weighted connectance in both years was correlated to sampling session (2020 – LMM, χ^2^ = 25.617, df = 6, *N* = 168, *p* < 0.001; 2021 – LMM, χ^2^ = 41.091, df = 5, *N* = 144, *p* < 0.001), but was not affected by fertilisation (2020 – LMM, χ^2^ = 0.589, df = 1, *N* = 168, *p* = 0.292; 2021 – LMM, χ^2^ = 0.027, df = 1, *N* = 144, *p* = 0.869). In the first year, the weighted connectance rose until the 4th session, during late stem elongation, then decreased towards the end of the season, while in the second year it decreased from the beginning until the fourth session, then slowly increased (Fig. 2).

### Specialisation

**Fig. 3:**
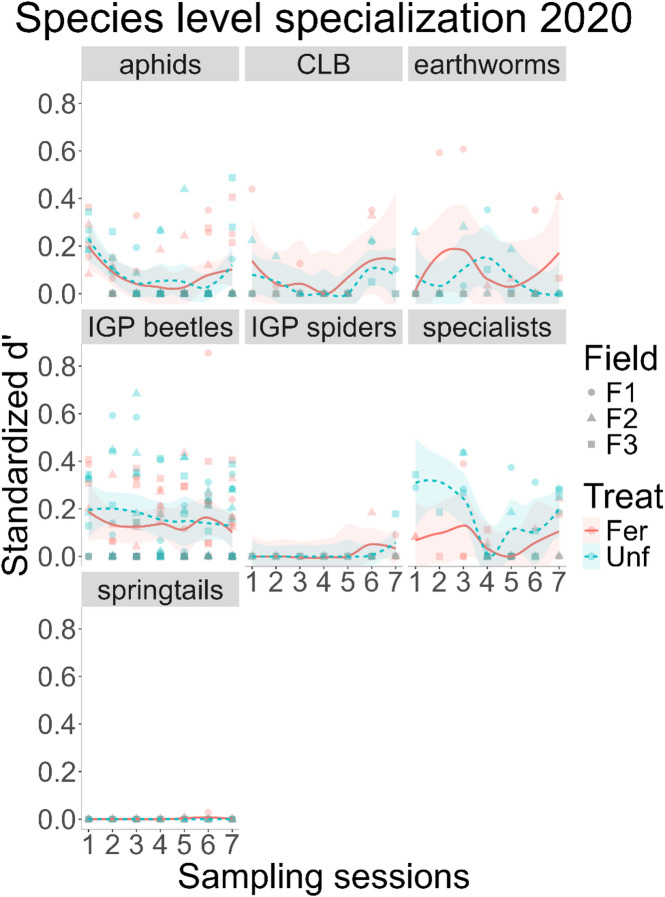
Species level specialisation (Blüthgen’s *d’*) across sampling sessions and fertilisation treatments, in 2020, by prey group. The lines correspond to loess smoothing, using the *geom_smooth* function of the *ggplot2* R package, with the standard error as the shaded areas. CLB – cereal leaf beetle (*Oulema melanopus*); IGP beetles – intraguild predation, corresponds to generalist predators consumed by sampled generalist predators; Specialists – aphid specialist predators consumed by sampled generalist predators.

The species level specialisation (*d’*^30^, , as metric of diet overlap by calculating the diversity of predators consuming a given prey, had different responses to fertilisation and time depending on the prey target. Starting with prey in 2020, the specialisation on aphids was affected by both fertilisation and session, as a joint or interaction effect (GLMM – χ^2^ = 14.479, df = 6, *N* = 109, *p* = 0.025), with the fertilised treatment having higher values towards the end of the season (Fig. 3). For the cereal leaf beetle, fertilisation had no effect on specialisation (GLM – F = 1.383, df = 1, *N* = 37, *p* = 0.249), but session did (GLM – F = 5.315, df = 6, *N* = 37, *p* < 0.001), with a slight increase towards the end of sampling (Fig. 3). Earthworm specialisation, on the other hand, was neither affected by fertilisation (GLM – F = 0.914, df = 1, *N* = 41, *p* = 0.346) nor session (GLM – F = 0.319, df = 6, *N* = 41, *p* = 0.922, Fig. 3). For springtails, 95% (40/42) of all *d’* values calculated were 0 (*d’* = 0 indicates a complete overlap on a given target), as a result, a model could not be estimated (Fig. 3).

Regarding intraguild prey, the specialisation on beetles was not affected by either fertilisation treatment (GLMM – χ^2^ = 0.937, df = 1, *N* = 158, *p* = 0.333), nor session (GLMM – χ^2^ = 7.559, df = 6, *N* = 158, *p* = 0.272, Fig. 3). As with beetles, specialisation on spiders was also not affected by fertilisation (GLM – F = 0.2995, df = 1, *N* = 30, *p* = 0.589) nor session (GLM - F = 2.050, df = 6, *N* = 30, *p* = 0.099, Fig. 3).

Lastly, for specialist predators, such as ladybugs and hoverflies, consumed by the generalist predator *taxa* sampled, the specialisation was once again not affected by fertilisation (GLM – F = 1.271, df = 1, *N* = 47, *p* = 0.266) nor session (GLM – F = 2.130, df = 6, *N* = 47, *p* = 0.071, Fig. 3).

**Fig. 4:**
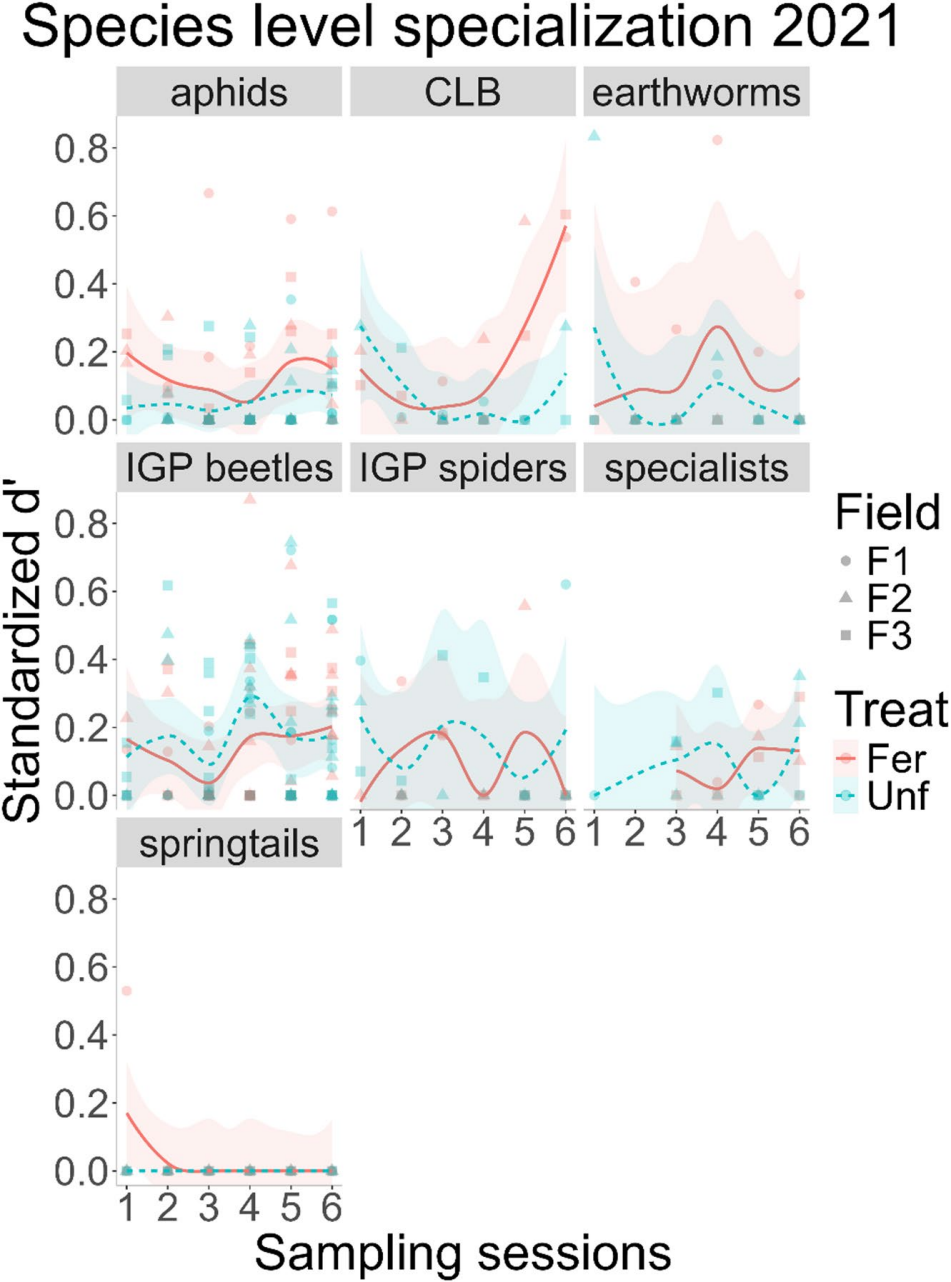
Species level specialisation (Blüthgen’s *d’*) across sampling sessions and fertilisation treatments, in 2021, by prey group. The lines correspond to loess smoothing, using the *geom_smooth* function of the *ggplot2* R package, with the standard error as the shaded areas. CLB – cereal leaf beetle (*Oulema melanopus*); IGP beetles – intraguild predation, corresponds to generalist predators consumed by sampled generalist predators; Specialists – aphid specialist predators consumed by sampled generalist predators.

Moving onto prey in 2021, specialisation on aphids was affected by fertilisation treatment (GLMM – χ^2^ = 6.054, df = 1, N = 92, p = 0.014) but not session (GLMM – χ^2^ = 5.258, df = 5, N = 92, p = 0.385), with fertilisation increasing specialisation (Fig. 4). On the cereal leaf beetle, both fertilisation (GLMM – Chi = 14.415, df = 5, N = 28, p = 0.013) and session (GLMM – Chi = 3.905, df = 1, N = 28, p = 0.048) had an effect on specialisation, with an inverse bell shape over time, with a pronounced increase in the fertilised treatment (Fig. 4). In contrast, earthworms were neither affected by fertilisation (GLM – F = 0.867, df = 1, N = 33, p = 0.359), nor session (GLM – F = 0.858, df = 5, N = 33, p = 0.522, Fig. 4). As in the previous year, 97% (35/36) of the springtails d’ values were 0, hence a model could not be estimated, but it once again points to this group being a staple food source for generalist predators (Fig. 4).

**Fig. 5:**
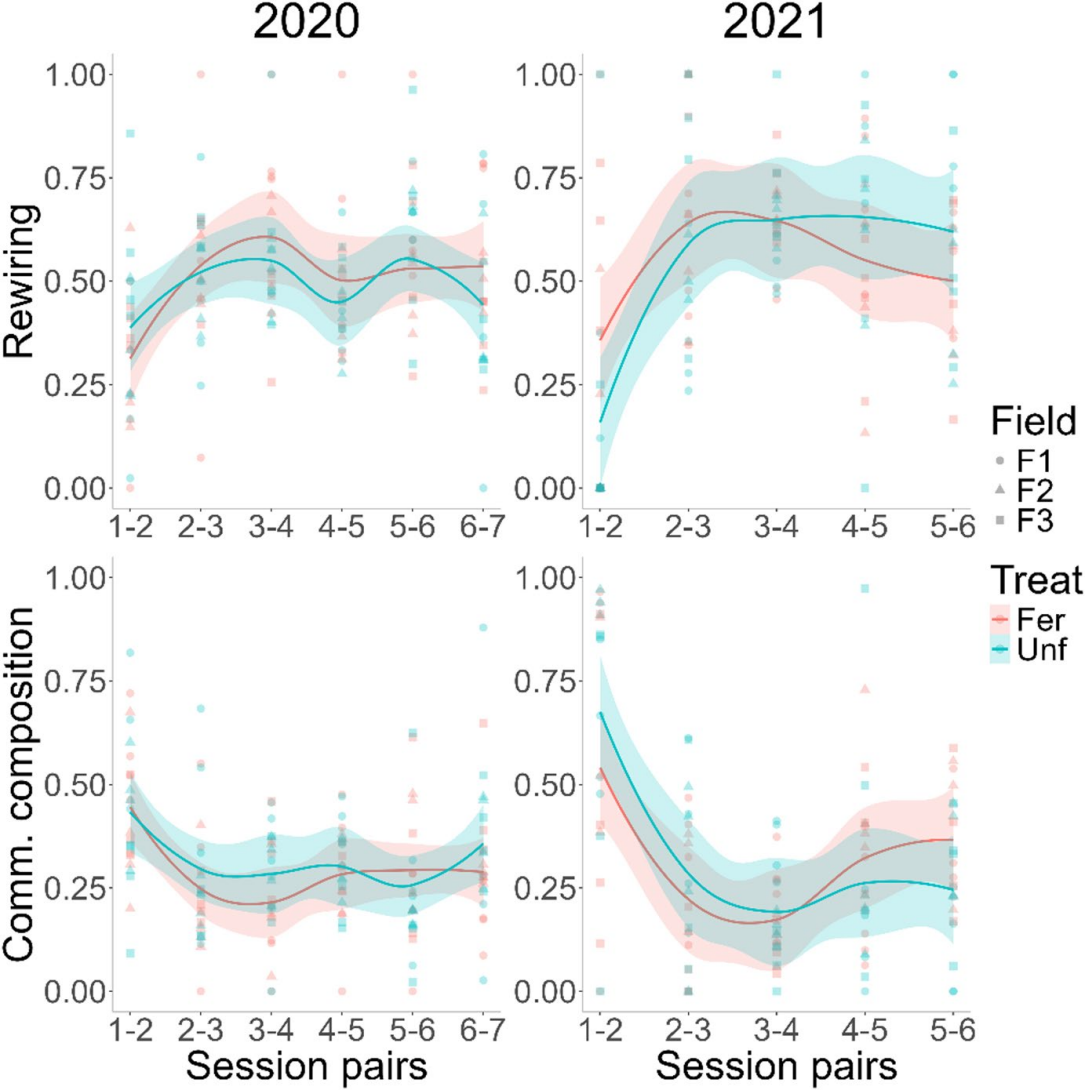
Food web dissimilarity across sampling sessions, for 2020 and 2021, respectively. Two indices were calculated, the trophic link rewiring and community dissimilarity using the *betalinkr* function from the *bipartite* R package. The lines correspond to loess smoothing, using the *geom_smooth* function of the *ggplot2* R package, with the standard error as the shaded areas.

Specialisation on beetles as intraguild prey was not significantly affected by fertilisation (GLMM – χ^2^ = 0.109, df = 1, *N* = 130, *p* = 0.740), as opposed to session (GLMM – χ^2^ = 14.326, df = 5, *N* = 130, *p* = 0.012), with a clear increase after the 3rd session (Fig. 5). For spiders, neither the treatment (GLM – F = 0.233, df = 1, *N* = 31, *p* = 0.634) nor the sampling session had an effect on specialisation (GLM – F = 0.323, df = 5, *N* = 31, *p* = 0.895, Fig. 4).

For specialist predators, neither fertilisation (GLM – F = 0.004, df = 1, *N* = 23, *p* = 0.951) nor session (GLM – F = 0.707, df = 4, *N* = 23, *p* = 0.597) had an effect on specialisation, though on the fertilised treatment there were no detections until the 3rd sampling session (Fig. 4).

### Trophic link rewiring and community composition dissimilarity

As with complexity, the fertilisation treatment did not affect the trophic link rewiring (2020 – LMM, χ^2^ = 0.199, df = 1, *N* = 143, *p* = 0.655; 2021 – LMM, χ^2^ = 0.007, df = 1, *N* = 116, *p* = 0.934). However, it changed over the season in both years (2020 – LMM, χ^2^ = 27.455, df = 5, *N* = 143, *p* < 0.001; 2021 – LMM, χ^2^ = 35.558, df = 4, *N* = 116, *p* < 0.001), by increasing in the first few sessions, then in 2020 alone it dipped during the mid-season, rising again in the end (Fig. 5).

Network community composition was similarly affected, once again changing across sampling sessions (2020 – LMM, χ^2^ = 26.919, df = 5, *N* = 143, *p* < 0.001; 2021 – LMM, χ^2^ = 45.448, df = 4, *N* = 116, *p* < 0.001), but not with fertilisation (2020 – LMM, χ^2^ = 0.662, df = 1, *N* = 143, *p* = 0.416; 2021 – LMM, χ^2^ = 0.021, df = 1, *N* = 116, *p* = 0.884) each year (Fig. 5). In both years, community composition dissimilarity dropped until the 3rd session, then for 2020 is stabilized, while it rose again in 2021, albeit slightly (Fig. 5).

In 2020, during the middle of the season, when aphids reached their peak (sessions 4 and 5^13^, the trophic links changed less as seen from the drop in rewiring to approximately 0.5 (Fig. 5). In 2021 the early rewiring (sessions 1 and 2) was less common, at approximately 0.2–0.3, although this appears to be the result of a few outliers (Fig. 5).

## Discussion

Our findings show how dynamic food web interactions within invertebrate communities in agro-ecosystems can be, even over very short time scales. Furthermore, we also show that over these short time scales, weighted connectance did not increase towards the mid-season peak of productivity, as expected in our first hypothesis, and instead decreased, in accordance with previous studies^28,29,32^. A low complexity may also indicate that there is not a strong selective pressure for predators to differentiate niches, which may be one of the reasons why we did not observe any strong fertilisation effects in our study.

The low complexity/selective pressure mentioned above could have a positive influence on the biological control services provided by generalist predators, considering that it may have allowed them greater freedom from competition, to exploit aphid and cereal leaf beetle prey^13,33^. As such, the regulation of pest prey was generalized across the predator community and therefore overlapping, only partially confirming our second hypothesis. However, this pattern was not evident for other prey. Of these, springtails seemingly have the role of a staple food, as they were very generally consumed by the predator community throughout the season. Furthermore, there is seemingly a conflict between specialist and generalist predators^34–36^, which consume both the specialists and their prey, in this case, the aphids. However, this may rather have a legacy effect on pest regulation, as specialists mostly appeared to be a generally consumed prey only after the peak aphid infestation.

We can furthermore show that rewiring within food webs, especially at the onset of the season, was increasing drastically until mid-season, as opposed to community composition, partially supporting our third hypothesis. As the fields grew taller and greener, more species came in and established interactions amongst themselves, then left at the end of the season; a pattern that has been observed before^37–40^. The centrality of different prey also shifted, with aphids becoming more central and accounting for larger proportions of the predators’ diets, precisely when their abundance reached its peak in the study fields. Meanwhile, ubiquitous prey, like springtails, remained central food sources throughout the entire sampling period.

Similarly, the food webs’ complexity changed over time, each year displaying a different trend, which may be linked to an aphid infestation that took place in 2020^13^; something which we discuss further down below. In terms of specialisation across prey, springtails were a widely consumed in both years, and the prevalence of maximum overlap is a strong indicator that springtails were a staple food source, for all predator *taxa* sampled. However, note that without an available measurement of consumed amounts, we cannot know whether this “stapleness” means they are a main source of energy, or just consumed ubiquitously in low quantities; posing an interesting question on how occurrence frequency correlates to consumption rates in field settings. Without that information, we cannot know whether the absence of changes in overlap in this study, or activity-density found by others^41^, correspond to prey switching, away from pests.

Spiders too appeared to be consumed by nearly all beetle species in 2020, pointing to a widespread occurrence of IGP. This did not take place in the following year, with fewer detections as well, probably as a result of a decline in spider abundance in the late season (Supplementary Fig. 15). As for predation on aphid specialist predators, the number of detections early in the season in both years is low, being altogether absent until the 3rd session in the fertilised treatment in 2021. These specialist predators follow aphid densities, lagging behind in their arrival to the fields and population growth^42^, hence this low number of detections was expected in the early season.

Regarding earthworms, their consumption appears to be sporadic; possibly due to them being low quality^43^ and, therefore, nonpreferred food sources when compared to other prey^23^, even for species known to consume them. In contrast to the earthworms, the specialisation on cereal leaf beetles was in line with their seasonal abundance^44^ for both years. Lastly, the specialisation on aphids poses an interesting case study. As mentioned above, there was a difference in aphid abundance across years^13^. The first year, with the infestation, specialisation on aphids showed a shallow inverted bell shape, which would be consistent with the peak in abundance. In contrast, there was no such effect in 2021, where specialisation was lower in the fertilised treatment.

Following that line of thought, we can gain further insight on the trends in community composition and rewiring dissimilarity seen in 2020 and 2021, once again leaning on the difference in prey abundance between years, caused by aphids. While phenology would mostly account for “when” species turnover, competition should account, at least partially, for their behaviour and by “how many” species are replaced. As such, the reduction of competition among predators, induced by the aphid infestation, possibly contributed to the shallower fluctuations in community composition seen in 2020. Likewise, for the trophic link rewiring, there was a brief period around the 4th and 5th sessions, of the same year, when predators diet changed less; this coincided with peak aphid abundance in the fields, and is reflected by their centrality in the food webs.

When looking at all the parameters measured in this study, most of them changed across time, in accordance with^7^. At a short, intra-annual scale, there were changes in rewiring, much like in long-term studies^45^, but we also observed a temporal variability for the weighted connectance that other studies did not^46^. Given the link between species richness and connectance^26,27^, this change can be explained by the species’ phenologies^47^. Such changes over a short period imply that biological control, as an ecosystem service, may fluctuate over time in effectiveness^14^. Adding to that, intraguild predation and general interference between generalist and specialist predators introduce another layer of complexity^48–51^. Moreover, we know that the services provided by certain species of hoverflies can change over time, depending on their life-cycle stage, for example going from predators to pollinators^52,53^.

Given the time frame of these changes, sampling the system without sufficient temporal replication within the same year, or once per year at the same period over multiple years, or locations, increases the likelihood of spatial or temporal uncoupling of species interactions^54^, leading to their underrepresentation or absence altogether. Furthermore, beyond the design and replication of sampling, there is also the matter of the sampling technique used, as it can have distinct biases and implications, not just for food webs, but ecological networks in general^54,55^. For the former in particular, the advantages and disadvantages of molecular analysis of food webs have been given considerable attention over the years (e.g^56–59^). However, among them, aspects such as the detection time of prey in the gut^60,61^, accounting for a wider time interval than direct observations, bear direct relevance for the interpretation of the data collected. Likewise, the inability to quantify consumption through molecular means, which may nonetheless be extremely difficult or near-impossible to do through observation for certain *taxa* in field studies^56,58^, are also particularly relevant for biological control.

Additionally, due to the fact this study was conducted in an open field setting, it is not possible to exclude the effects of flying prey and weed seeds^62^ as food sources. These food sources may affect parameters such as weighted connectance and rewiring, and in some cases shift the focus of predators away from potential pests^63^. Alternatively, it may further help reduce competition and allow for a greater overlap of predator diet. However, that was beyond the scope of the sampling and analysis effort of this study. Therefore, we limit our conclusions to the prey that were targeted within our study, some of which can fly or otherwise move through the air at some stage of their lifecycle (e.g. aphids, ballooning spiders, lacewings, hoverflies, lady bugs, etc.).

## Conclusion

Quantitative assessments of consumption, how plastic food webs are, and how niches change over time, are likely to be of key importance for building a more in depth understanding of theoretical and empirical food webs. This will allow us to explore the finer details of food webs, without which it will be difficult to identify nodes that are either central within food webs or account for a considerable portion of the energy requirements of predators. These nodes are keystones of the food webs, and what we can show here is that how central each prey is within food webs is something that can, and does, change very quickly. Some prey are, for example, central in food webs only for a limited period of time, such as outbreak or pest species, whereas others are central throughout the season, such as staple food sources (e.g. springtails).

The capacity of different prey (either as staple foods or as temporarily available resources) to sustain the predator community and affect predator species’ behaviour and competition, is also likely to play a large role in shaping food webs. That food web rewiring, in general, was greater than species turnover supports this, and indicates that food web changes, to a great extent, are due to shifts in behaviour, rather than species turnover. Additionally, the considerable short-term dynamics of our food webs, coupled with the tendency of most studies on ecosystem services to sample when services are needed (i.e. during the peak of the crop season), implies that the dynamic nature of food webs could be one of the reasons for why studies on biological control have produced inconsistent results^42,64^. Parameters, such as specialisation, have been overestimated, due to the inevitability of incomplete sampling of empirical networks^54,65^. For the same reasons, it may also be that we systematically have either underestimated, or overestimated ecosystem functioning, depending on when sampling has occurred. This ought to be considered when sampling or modelling such systems, or when attempting to manage them to strengthen ecosystem services, such as biological control.

## Methods

### Study site

**Fig. 6:**
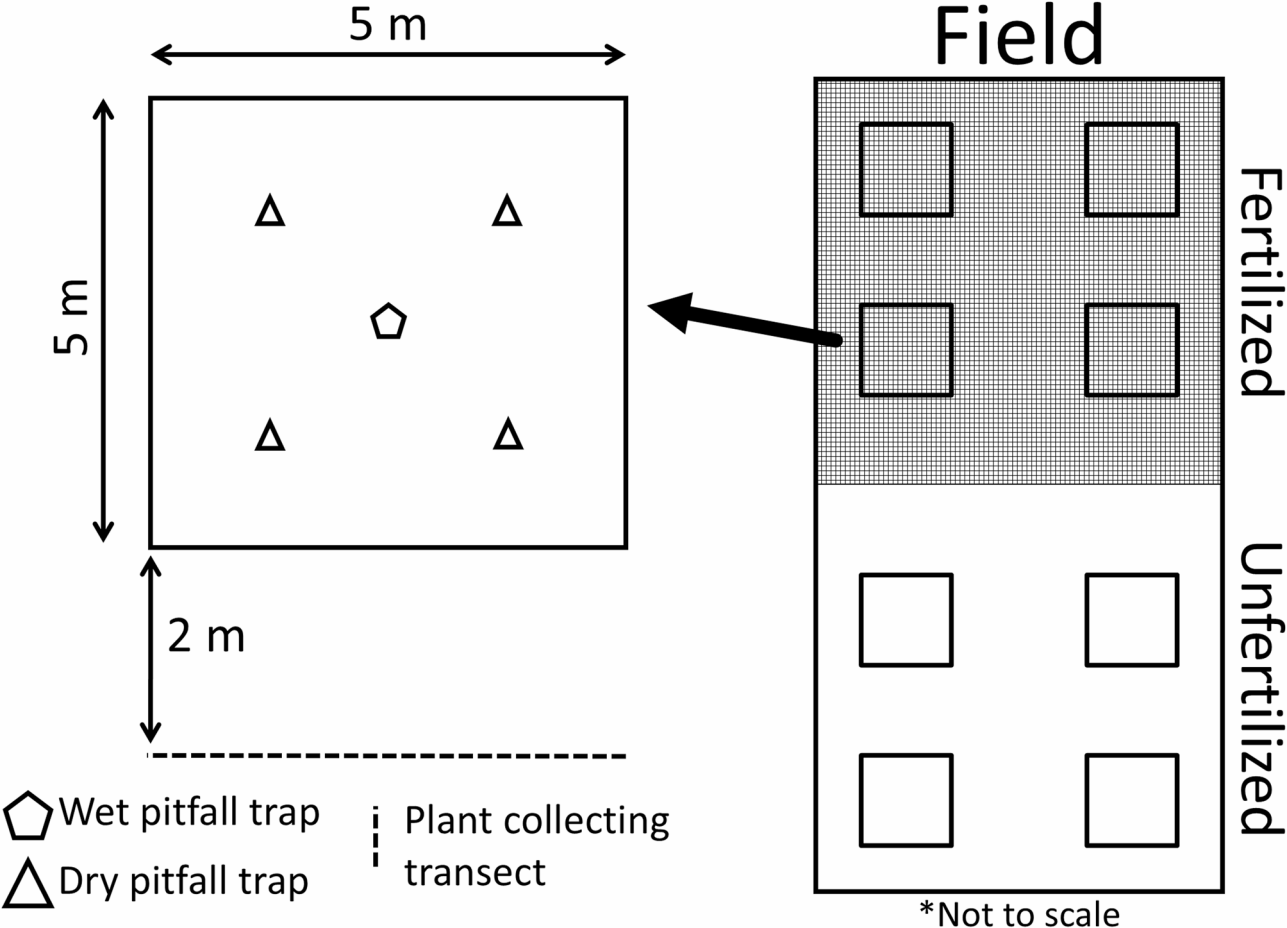
Field and sampling plot schematic (not to scale). Each field had one half of its area fertilised with manure at rate of 1 500 kg/ha, and the other half was left untreated as a control, with no physical barriers between treatments (e.g. no snail fences). Community sampling was done through wet pitfall trap sampling, while live predators were collected with dry pitfall traps to sample their diets. Plant collecting transects were done alongside the margins of the sampling plots, to count the number of aphids per cereal tiller in the fields. Sampling plots were at least 5 m from the field edge to avoid edge effects, 10 m way from one another along the field’s width, and between 15 to 20 m along the length of the field. To avoid contamination of the fertilisation effect, plots were at least 25 m away from the border between the treatments within a field.

The study site was in Kematen in Tirol, Austria, where spring barley (*Hordeum vulgare* L.) was grown in six organically managed fields, three in 2020 and three in 2021. The fields were tilled, pressed and fertilised prior to sowing, between March and April, and the barley ripened around late July to early August, after which point it was harvested. Before sowing, each field was split and one half was fertilised with cattle manure and the other remaining unfertilised, as a control. The manure was applied independently by each field*’*s respective owner, at a rate of 1 500 kg per hectare, using manure spreaders. For each treatment, four 5 × 5 m sampling plots were drawn (Fig. 6), with no barriers (e.g. cages or snail fences^66^, within the fields. Sampling plots were at least 5 m from the field edge to avoid edge effects, 10 m way from one another along the field’s width, and between 15 and 20 m along the length of the field. To avoid contamination of the fertilisation effect, plots were at least 25 m away from the border between the treatments within a field.

### Sampling methods

Sampling was conducted every two weeks, between the 21st of April and the 14th of July in 2020, and the 3rd of May and the 12th of July in 2021, and all fields in each year were sampled on the same week. Wet pitfall traps were active for four days, with 5–6 g of salt and 1mL of detergent per litre of water, to sample the soil surface community (Supplementary Tables 1 to 3). Dry pitfall traps were active for a single day, with wood chips within, to catch live predators to obtain gut content for molecular dietary analysis^67^, Supplementary Tables 4 to 6). These predators were ground (Carabidae) and rove (Staphylinidae) beetles, and spiders (Araneae). Additionally, transects were carried out along a single side of each plot, on the outside border, to avoid trampling on the inside. Thirty 30 individual barley plant tillers were collected to count the number of aphids per tiller for community analysis^13^.

### Molecular analysis

We captured a total of 2404 ground beetles, 913 rove beetles and 567 spiders in 2020, and 1977 ground beetles, 891 rove beetles and 250 spiders in 2021 (Supplementary Tables 7 and 8, respectively). The beetles’ gut content and the spiders’ full bodies were extracted using a BioSprint 96 DNA Blood Kit (Qiagen, Hilden, Germany) on a QIAGEN Biosprint96^®^ workstation, following the manufacturer’s recommendations. Each sample was analysed three times, with different multiplex-PCR assays. The first focused on several prey *taxa* (assay in^68^, earthworms, aphids, springtails and the cereal leaf beetle. The second targeted generalist and specialist predators, such as spiders, lacewings and ladybeetles (primers from^67,69^. The third assay identified the genus of beetles consumed, from a selected set of common *taxa* consisting of *Bembidion* spp., *Harpalus* spp., *Poecilus* spp., *Pterostichus* spp., *Philonthus carbonarius* and *Philonthus cognatus*.

The molecular gut content analysis is described in greater detail in^13^, but the multiplex PCR assays can also be found here, in the supplementary materials (Supplementary Tables 4 to 6), for convenience.

### Data analysis

All data analysis was carried out using R 4.1.2^70^ and RStudio 2023.03.1 + 446^71^, with the packages *lme4*^72^ for linear mixed effects modelling (LMM), *glmmTMB*^73^ for generalised linear mixed effects modelling (GLMM) and the *glm* function from base R for generalised linear modelling (GLM). The package *bipartite*^74,75^ was used for food web generation, and the calculation of several metrics: species level specialisation^30^, trophic link rewiring and community composition dissimilarity^31^, and weighted connectance^25^; using the mean proportion of diet detections as weights; Table 2). The package *tidyverse*^76^ was used for data processing, as well as to provide greater reproducibility of the data matrices obtained from the archived raw data, and *ggplot2*^77^ was used for graphic creation, for the same reasons. Plots with loess smoothing were created using the standard loess method from the *geom_smooth* function from *ggplot2*, and standard error as the shaded ribbons.

**Table 2:** Variables measured, with their respective calculations and what they represent in the context of this study.

Variable	Calculation	Representation
Weighted connectance	Link density (trophic links per species) divided by species, using mean proportion of diet detections as weights	Food web complexity
Rewiring dissimilarity	Food web dissimilarity explained by rewiring of trophic links (for the shared species subweb)	Trophic link dissimilarity
Community dissimilarity	Food web dissimilarity explained by species turnover	Community turnover
Species-level specialisation	Standardized predator diversity for each prey species	Predator dietary overlap

All these metrics were calculated based on the diet data matrices obtained from the diet analysis of ground (Carabidae) and rove (Staphylinidae) beetles, and spiders (Araneae).

For dietary data, detections were averaged for all individuals of a given species in each unique combination of plot, treatment, field and sampling session; resulting in a mean proportion of the detections. As an example, the diet proportions for the species *Poecilus cupreus* in plot 1, fertilised treatment, field 1 on sampling session 1 was the mean of all *P.cupreus* individual diet detections; which were recorded either as 1 – detected, or 0 – not detected, for each diet target in the assays (Tables 3 and 4). All self-detections (*e.g. P. cupreus* testing positive for *Poecilus* in PCR assay 3) were manually removed (setting the detection to 0, instead of 1).

**Table 3:** Example diet data matrix, with three individuals *Poecilus cupreus*, two captured in one sample, and one in another, each with a single prey target detection (A and B).

Session	Field	Treatment	Plot	Species	Prey A	Prey B
1	1	Fertilised	1	*P. cupreus*	0	1
1	1	Fertilised	1	*P. cupreus*	1	0
1	1	Fertilised	2	*P. cupreus*	1	0

**Table 4:** Example of diet matrix above, after calculating mean averages.

Session	Field	Treatment	Plot	Species	Prey A	Prey B
1	1	Fertilised	1	*P. cupreus*	0.5	0.5
1	1	Fertilised	2	*P. cupreus*	1	0

By calculating the average mean diet of the species *P. cupreus* for a sample (unique combination of sampling session, field, treatment and plot), we obtained the mean dietary proportion for that predator species in its respective sample, which was then used as weights for the calculation of weighted connectance.

Prior to testing, the heteroscedasticity of models was checked through visual inspection of quantile-quantile and residual vs. predicted plots^78^. The error structures of GLMMs and GLMs with proportion data were adapted to ordered beta (hence the use of *glmmTMB*, for access to the *ordbeta* family, which was not available in *lme4*), and quasibinomial (*quasibinomial* family in *glm*), respectively.

Due to the experimental design of our study, the experimental units were the fields, thus the sampling plots within them represent pseudoreplicates. In order to address the correlation among the plots in the same field, and minimize the likelihood of falsely detecting significant differences, we followed the method in^79^. By using mixed effects models with field (replicate-level grouping variable) as a random effect, it allowed us to correct for type I error and address the pseudoreplication bias.

In order to explicitly test the hypothesis of our study within our models, we set up appropriate contrasts for each variable. The session variable, being categorical, had a sliding contrast so that each session would be directly compared to the previous one; fertilisation had a treatment contrast with the unfertilised, or control, treatment as the baseline; while field, used as a random factor, had a sum contrast, so that the grand mean of all fields would be the reference value, as opposed any individual one.

The significance of the variables and interaction terms was assessed using the *anova* function of base R, through Chi-squared (χ^2^) tests for the mixed effects models, or with F tests for the GLMs, with the threshold defined at 0.05.

Three sets of linear mixed effects models (LMM) were created for each year, to test the effects of time and fertilisation on different variables. Our first model analysed how sampling session and fertilisation affected the weighted connectance of the food webs, with field as a random factor. We chose weighted connectance, which is the proportion of realized trophic links in a food web, as a metric for complexity, while factoring in weights. Unlike other complexity metrics (e.g. link density or unweighted connectance), it takes into account the strength of the trophic links. Doing so allows for a better understanding of predator behaviour and ecosystem function^80^, fitting in with the other metrics chosen (dissimilarity and specialisation) and the biological control setting of the project. The second and third models looked at how session and fertilisation affected the rewiring dissimilarity and community composition dissimilarity, respectively, with field as a random factor. These two components of dissimilarity were chosen as they allowed us to measure and compare how the food webs changed in structure over time. The rewiring component represents changes in behaviour within a subset of community that is common to both food webs (e.g. the changes in interactions between species present in both sampling session 1 and 2), while the community composition component represents species turnover (e.g. changes in species between food webs in sampling session 1 and 2).

The species level specialisation (*d*’) measured the diversity of predators consuming a given prey (e.g. predators consuming springtails), providing us with a metric of predator diet overlap, from the perspective of the prey target. It was chosen as it was complementary to the network level specialisation analysis (*H2’*, representing the overlap of the overall food webs), that we carried out in a previous paper^14^, and the other behavioural metrics described above. The analysis of specialisation for each prey target was carried out with generalised linear mixed effects models (GLMM), or with generalised linear models (GLM) when the number of diet detections was too low to allow fitting mixed effect models. In both cases, the models tested the effects of sampling session and fertilisation on the standardized *d’*, with the GLMMs using the sampling field as a random factor. In total there were seven models for each year, GLMMs for aphid and IGP beetle *d’*, and GLMs for cereal leaf beetle, earthworms, specialists, IGP spiders. For the springtails in particular, neither GLMMs nor GLMs worked, as over 95% of samples returned a *d’* of zero, thus it was not possible to fit any models.

## Supplementary Information

Below is the link to the electronic supplementary material.

Supplementary Material 1

## Data Availability

All data has been archived in the repository Figshare (https://figshare.com/), under the DOI: https://doi.org/10.6084/m9.figshare.26893354, https://doi.org/10.6084/m9.figshare.26893405, https://doi.org/10.6084/m9.figshare.26893624, https://doi.org/10.6084/m9.figshare.26893630, https://doi.org/10.6084/m9.figshare.26893690, https://doi.org/10.6084/m9.figshare.26893759.

## References

[1] Gadelha, Y. E., Lange, D., Dattilo, W. & Lopes, B. C. Phenological phases of the host plant shape plant–treehopper interaction networks. *Ecol. Entomol.* **42** (6), 827–837. 10.1111/een.12457 (2017).

[2] Morente-López, J., Lara-Romero, C., Ornosa, C. & Iriondo, J. M. Phenology drives species interactions and modularity in a plant-flower visitor network. *Sci. Rep.* **8** (1), 9386. 10.1038/s41598-018-27725-2 (2018). 29925965 10.1038/s41598-018-27725-2 PMC6010405

[3] Foley, J. A. et al. Solutions for a cultivated planet. *Nature* **478** (7369), 337–342. 10.1038/nature10452 (2011). 21993620 10.1038/nature10452

[4] Mouillot, D., Graham, N. A., Villéger, S., Mason, N. W. & Bellwood, D. R. A functional approach reveals community responses to disturbances. *Trends Ecol. Evol.* **28** (3), 167–177. 10.1016/j.tree.2012.10.004 (2013). 23141923 10.1016/j.tree.2012.10.004

[5] Frainer, A., McKie, B. G. & Malmqvist, B. When does diversity matter? Species functional diversity and ecosystem functioning across habitats and seasons in a field experiment. *J. Anim. Ecol.* **83** (2), 460–469. 10.1111/1365-2656.12142 (2014). 26046457 10.1111/1365-2656.12142

[6] Zadoks, J. C., Chang, T. T. & Konzak, C. F. A decimal code for the growth stages of cereals. *Weed Res.* **14** (6), 415–421. 10.1111/j.1365-3180.1974.tb01084.x (1974).

[7] Thompson, R. M. & Townsend, C. R. *The Effect of Seasonal Variation on the Community Structure and food-web Attributes of Two Streams: Implications for food-web Science* 75–88 (Oikos, 1999). 10.2307/3546998

[8] Terry, J. C. D., Morris, R. J. & Bonsall, M. B. Trophic interaction modifications: an empirical and theoretical framework. *Ecol. Lett.* **20** (10), 1219–1230. 10.1111/ele.12824 (2017). 28921859 10.1111/ele.12824 PMC6849598

[9] Kaartinen, R. & Roslin, T. High Temporal consistency in quantitative food web structure in the face of extreme species turnover. *Oikos* **121** (11), 1771–1782. 10.1111/j.1600-0706.2012.20108.x (2012).

[10] Cuff, J. P., Windsor, F. M., Tercel, M. P., Kitson, J. J. & Evans, D. M. Overcoming the pitfalls of merging dietary metabarcoding into ecological networks. *Methods Ecol. Evol.* **13** (3), 545–559. 10.1111/2041-210X.13796 (2022).

[11] Cuff, J. P. et al. The predator problem and PCR primers in molecular dietary analysis: swamped or silenced; depth or breadth? *Mol. Ecol. Resour.* **23** (1), 41–51. 10.1111/1755-0998.13705 (2023). 36017818 10.1111/1755-0998.13705 PMC10087656

[12] Deagle, B. E., Pansu, J., McInnes, J. & Traugott, M. Revealing animal diet and food webs through DNA metabarcoding. In *Applied Environmental Genomics* (eds. Jarman, S., Holleley, C. & Berry, O.) 30–45 (CSIRO Publishing, 2023).

[13] Leote, P. N. B., Rubbmark, O. R. R. & Traugott, M. High resolution Temporal data shows how increasing prey availability reduces early season intraguild predation and pest spread in cereal crops. *Biol. Control*. 105549. 10.1016/j.biocontrol.2024.105549 (2024).

[14] Branco Leote, P. N., Rubbmark, R., Traugott, M. & O. R., & Molecular assessment of food web dynamics identifies critical periods for managing resilience in biological pest control. *Ecol. Appl.* **35** (5), e70078. 10.1002/eap.70078 (2025). 40744864 10.1002/eap.70078 PMC12313443

[15] Rowen, E., Tooker, J. F. & Blubaugh, C. K. Managing fertility with animal waste to promote arthropod pest suppression. *Biol. Control*. **134**, 130–140. 10.1016/j.biocontrol.2019.04.012 (2019).

[16] Van Emden, H. F. & Harrington, R. (eds) *Aphids as Crop Pests* (Cabi, 2017).

[17] Van de Vijver, E. et al. Inter-and intrafield distribution of cereal leaf beetle species (Coleoptera: Chrysomelidae) in Belgian winter wheat. *Environ. Entomol.* **48** (2), 276–283. 10.1111/eea.12835 (2019). 30715239 10.1093/ee/nvz002

[18] Sunderland, K. D. & Vickerman, G. P. Aphid feeding by some polyphagous predators in relation to aphid density in cereal fields. *J. Appl. Ecol.* 389–396. 10.2307/2402334 (1980).

[19] Holopainen, J. K. & Helenius, J. Gut contents of ground beetles (Col., Carabidae), and activity of these and other epigeal predators during an outbreak of rhopalosiphum Padi (Hom., Aphididae). *Acta Agriculturae Scand. B-Plant Soil. Sci.* **42** (1), 57–61. 10.1080/09064719209410199 (1992).

[20] Thies, C. et al. The relationship between agricultural intensification and biological control: experimental tests across Europe. *Ecol. Appl.* **21** (6), 2187–2196. 10.1890/10-0929.1 (2011). 21939053 10.1890/10-0929.1

[21] Kheirodin, A., Sharanowski, B. J., Cárcamo, H. A. & Costamagna, A. C. Consumption of cereal leaf beetle, Oulema melanopus, by generalist predators in wheat fields detected by molecular analysis. *Entomol. Exp. Appl.* **168** (1), 59–69. 10.1111/eea.12835 (2020).

[22] Snyder, W. E. & Ives, A. R. Interactions between specialist and generalist natural enemies: parasitoids, predators, and pea aphid biocontrol. *Ecology* **84** (1), 91–107. 10.1890/0012-9658( (2003). 2003)084[0091:IBSAGN]2.0.CO;2.

[23] Symondson, W. O. C., Glen, D. M., Erickson, M. L., Liddell, J. E. & Langdon, C. J. Do earthworms help to sustain the slug predator pterostichus melanarius (Coleoptera: Carabidae) within crops? Investigations using monoclonal antibodies. *Mol. Ecol.* **9** (9), 1279–1292. 10.1046/j.1365-294x.2000.01006.x (2000). 10972768 10.1046/j.1365-294x.2000.01006.x

[24] Eitzinger, B. & Traugott, M. Which prey sustains cold-adapted invertebrate generalist predators in arable land? Examining prey choices by molecular gut‐content analysis. *J. Appl. Ecol.* **48** (3), 591–599. 10.1111/j.1365-2664.2010.01947.x (2011).

[25] Bersier, L. F., Banašek-Richter, C. & Cattin, M. F. Quantitative descriptors of food-web matrices. *Ecology* **83** (9), 2394–2407. 10.1890/0012-9658(2002 (2002). )083[2394:QDOFWM]2.0.CO;2.

[26] Banašek-Richter, C. et al. Complexity in quantitative food webs. *Ecology* **90** (6), 1470–1477. 10.1890/08-2207.1 (2009). 19569361 10.1890/08-2207.1

[27] Calizza, E., Rossi, L., Careddu, G., Caputi, S., Costantini, M. L. & S., & Species richness and vulnerability to disturbance propagation in real food webs. *Sci. Rep.* **9** (1), 19331. 10.1038/s41598-019-55960-8 (2019). 31852953 10.1038/s41598-019-55960-8 PMC6920442

[28] Worm, B. & Duffy, J. E. Biodiversity, productivity and stability in real food webs. *Trends Ecol. Evol.* **18** (12), 628–632. 10.1016/j.tree.2003.09.003 (2003).

[29] Nie, S. et al. Will a large complex system be productive? *Ecol. Lett.* **26** (8), 1325–1335. 10.1111/ele.14242 (2023). 37190868 10.1111/ele.14242

[30] Blüthgen, N., Menzel, F. & Blüthgen, N. Measuring specialisation in species interaction networks. *BMC Ecol.* **6** (1), 1–12. 10.1186/1472-6785-6-9 (2006). 16907983 10.1186/1472-6785-6-9 PMC1570337

[31] Poisot, T., Canard, E., Mouillot, D., Mouquet, N. & Gravel, D. The dissimilarity of species interaction networks. *Ecol. Lett.* **15** (12), 1353–1361. 10.1111/ele.12002 (2012). 22994257 10.1111/ele.12002

[32] Parker, S. M. & Huryn, A. D. Disturbance and productivity as codeterminants of stream food web complexity in the Arctic. *Limnol. Oceanogr.* **58** (6), 2158–2170. 10.4319/lo.2013.58.6.2158 (2013).

[33] Michalko, R., Pekár, S. & Entling, M. H. An updated perspective on spiders as generalist predators in biological control. *Oecologia* **189**, 21–36. 10.1007/s00442-018-4313-1 (2019). 30535723 10.1007/s00442-018-4313-1

[34] Rosenheim, J. A., Limburg, D. D. & Colfer, R. G. Impact of generalist predators on a biological control agent, Chrysoperla carnea: direct observations. *Ecol. Appl.* **9** (2), 409–417. 10.1890/1051-0761(1999)009[0409:IOGPOA]2.0.CO;2 (1999).

[35] Snyder, W. E. & Ives, A. R. Generalist predators disrupt biological control by a specialist parasitoid. *Ecology* **82** (3), 705–716. 10.1890/0012-9658(2001)082 (2001). [0705:GPDBCB]2.0.CO;2.

[36] Diehl, E., Sereda, E., Wolters, V. & Birkhofer, K. Effects of predator specialization, host plant and climate on biological control of aphids by natural enemies: a meta-analysis. *J. Appl. Ecol.* **50** (1), 262–270. 10.1111/1365-2664.12032 (2013).

[37] Collins, K. L., Boatman, N. D., Wilcox, A., Holland, J. M. & Chaney, K. Influence of beetle banks on cereal aphid predation in winter wheat. *Agric. Ecosyst. Environ.* **93** (1–3), 337–350. 10.1016/S0167-8809(01)00340-1 (2002).

[38] Thomas, C. G., Holland, J. M. & Brown, N. J. *The Spatial Distribution of Carabid Beetles in Agricultural Landscapes* 305–344 (The agroecology of carabid beetles, 2002).

[39] Holland, J. M. et al. The Spatial dynamics and movement of pterostichus melanarius and P. madidus (Carabidae) between and within arable fields in the UK. *Int. J. Ecol. Environ. Sci.* **30**, 35–53 (2004).

[40] Öberg, S. & Ekbom, B. Recolonisation and distribution of spiders and carabids in cereal fields after spring sowing. *Ann. Appl. Biol.* **149** (2), 203–211. 10.1111/j.1744-7348.2006.00088.x (2006).

[41] Birkhofer, K., Wise, D. H. & Scheu, S. Subsidy from the detrital food web, but not microhabitat complexity, affects the role of generalist predators in an aboveground herbivore food web. *Oikos* **117** (4), 494–500. 10.1111/j.0030-1299.2008.16361.x (2008).

[42] Raymond, L., Ortiz-Martínez, S. A. & Lavandero, B. Temporal variability of aphid biological control in contrasting landscape contexts. *Biol. Control*. **90**, 148–156. 10.1016/j.biocontrol.2015.06.011 (2015).

[43] Fawki, S., Smerup, S. & Toft, S. Food preferences and food value for the carabid beetles Pterostichus melanarius, P. versicolor and Carabus nemoralis. In Proceedings of the 11th European Carabidologist Meeting (pp. 99–109). (2005), January.

[44] McPherson, R. M. Seasonal abundance of cereal leaf beetles (Coleoptera: Chrysomelidae) in Virginia small grains and corn. *J. Econ. Entomol.* **76** (6), 1269–1272. 10.1093/jee/76.6.1269 (1983).

[45] Olesen, J. M., Stefanescu, C. & Traveset, A. Strong, long-term Temporal dynamics of an ecological network. *PloS One*. **6** (11), e26455. 10.1371/journal.pone.0026455 (2011). 22125597 10.1371/journal.pone.0026455 PMC3219636

[46] Trøjelsgaard, K. & Olesen, J. M. Ecological networks in motion: micro-and macroscopic variability across scales. *Funct. Ecol.* **30** (12), 1926–1935. 10.1111/1365-2435.12710 (2016).

[47] Suzuki, S. S., Baba, Y. G. & Toju, H. Dynamics of species-rich predator–prey networks and seasonal alternations of core species. *Nat. Ecol. Evol.* **7** (9), 1432–1443. 10.1038/s41559-023-02130-9 (2023). 37460838 10.1038/s41559-023-02130-9

[48] Hindayana, D., Meyhöfer, R., Scholz, D. & Poehling, H. M. Intraguild predation among the hoverfly episyrphus balteatus de Geer (Diptera: Syrphidae) and other aphidophagous predators. *Biol. Control*. **20** (3), 236–246. 10.1006/bcon.2000.0895 (2001).

[49] Janssen, A. et al. Intraguild predation usually does not disrupt biological control. In *Trophic and Guild Interactions in Biological Control (ed. Brodeur, J. & Boivin, G.)* 21–44 (Springer Netherlands, 2006).

[50] Lucas, É. & Rosenheim, J. A. Influence of extraguild prey density on intraguild predation by heteropteran predators: A review of the evidence and a case study. *Biol. Control*. **59** (1), 61–67. 10.1016/j.biocontrol.2011.05.010 (2011).

[51] Liang, Y. et al. Flower provision reduces intraguild predation between predators and increases aphid biocontrol in tomato. *J. Pest Sci.* **95** (1), 461–472. 10.1007/s10340-021-01396-x (2022).

[52] Dunn, L., Lequerica, M., Reid, C. R. & Latty, T. Dual ecosystem services of syrphid flies (Diptera: Syrphidae): pollinators and biological control agents. *Pest Manag. Sci.* **76** (6), 1973–1979. 10.1002/ps.5807 (2020). 32115861 10.1002/ps.5807

[53] Rodríguez-Gasol, N., Alins, G., Veronesi, E. R. & Wratten, S. The ecology of predatory hoverflies as ecosystem-service providers in agricultural systems. *Biol. Control*. **151**, 104405. 10.1016/j.biocontrol.2020.104405 (2020).

[54] Jordano, P. Sampling networks of ecological interactions. *Funct. Ecol.* **30** (12), 1883–1893. 10.1111/1365-2435.12763 (2016).

[55] Dormann, C. F., Fründ, J. & Schaefer, H. M. Identifying causes of patterns in ecological networks: opportunities and limitations. *Annu. Rev. Ecol. Evol. Syst.* **48** (1), 559–584. 10.1146/annurev-ecolsys-110316-022928 (2017).

[56] Symondson, W. O. C. Molecular identification of prey in predator diets. *Mol. Ecol.* **11** (4), 627–641. 10.1046/j.1365-294X.2002.01471.x (2002). 11972753 10.1046/j.1365-294x.2002.01471.x

[57] King, R. A., Read, D. S., Traugott, M. & Symondson, W. O. C. INVITED REVIEW: molecular analysis of predation: a review of best practice for DNA-based approaches. *Mol. Ecol.* **17** (4), 947–963. 10.1111/j.1365-294X.2007.03613.x (2008). 18208490 10.1111/j.1365-294X.2007.03613.x

[58] Clare, E. L. Molecular detection of trophic interactions: emerging trends, distinct advantages, significant considerations and conservation applications. *Evol. Appl.* **7** (9), 1144–1157. 10.1111/eva.12225 (2014). 25553074 10.1111/eva.12225 PMC4231602

[59] Symondson, W. O. C. & Harwood, J. D. Special issue on molecular detection of trophic interactions: unpicking the tangled bank. *Mol. Ecol.* **23** (15), 3601–3604. 10.1111/mec.12831 (2014). 25051891 10.1111/mec.12831

[60] Greenstone, M. H., Rowley, D. L., Weber, D. C., Payton, M. E. & Hawthorne, D. J. Feeding mode and prey detectability half-lives in molecular gut-content analysis: an example with two predators of the Colorado potato beetle. *Bull. Entomol. Res.* **97** (2), 201–209. 10.1017/S000748530700497X (2007). 17411483 10.1017/S000748530700497X

[61] Fülöp, D., Szita, É., Gerstenbrand, R., Tholt, G. & Samu, F. Consuming alternative prey does not influence the DNA detectability half-life of pest prey in spider gut contents. *PeerJ* **7**, e7680. 10.7717/peerj.7680 (2019). 31660259 10.7717/peerj.7680 PMC6814063

[62] Neidel, V., Vašková, H., Wallinger, C. & Saska, P. Variation in weed seed DNA detectability among arable carabids with different trophic specialization. *Arthropod-Plant Interact.* **19** (1), 19. 10.1007/s11829-024-10122-0 (2025).

[63] Mundy, C. A., Allen-Williams, L. J., Underwood, N. & Warrington, S. Prey selection and foraging behaviour by pterostichus cupreus L.(Col., Carabidae) under laboratory conditions. *J. Appl. Entomol.* **124** (9‐10), 349–358. 10.1046/j.1439-0418.2000.00491.x (2000).

[64] Karp, D. S. et al. Crop pests and predators exhibit inconsistent responses to surrounding landscape composition. Proceedings of the National Academy of Sciences, 115(33), E7863-E7870. (2018). 10.1073/pnas.1800042115 10.1073/pnas.1800042115 PMC6099893 30072434

[65] Fründ, J., McCann, K. S. & Williams, N. M. Sampling bias is a challenge for quantifying specialization and network structure: lessons from a quantitative niche model. *Oikos* **125** (4), 502–513. 10.1111/oik.02256 (2016).

[66] Staudacher, K. et al. Habitat heterogeneity induces rapid changes in the feeding behaviour of generalist arthropod predators. *Funct. Ecol.* **32** (3), 809–819. 10.1111/1365-2435.13028 (2018). 29657351 10.1111/1365-2435.13028 PMC5887929

[67] Staudacher, K., Jonsson, M. & Traugott, M. Diagnostic PCR assays to unravel food web interactions in cereal crops with focus on biological control of aphids. *J. Pest Sci.* **89**, 281–293. 10.1007/s10340-015-0685-8 (2016). 10.1007/s10340-015-0685-8 PMC4757624 26924957

[68] Rennstam Rubbmark, O., Sint, D., Cupic, S. & Traugott, M. When to use next generation sequencing or diagnostic PCR in diet analyses. *Mol. Ecol. Resour.* **19** (2), 388–399. 10.1111/1755-0998.12974 (2019). 30506979 10.1111/1755-0998.12974 PMC6446722

[69] Sint, D., Niederklapfer, B., Kaufmann, R. & Traugott, M. Group-specific multiplex PCR detection systems for the identification of flying insect prey. *PloS One*. **9** (12), e115501. 10.1371/journal.pone.0115501 (2014). 25525799 10.1371/journal.pone.0115501 PMC4272292

[70] R Core Team. R: A Language and Environment for Statistical Computing. R Foundation for Statistical Computing, Vienna, Austria. (2024). https://www.R-project.org/

[71] Posit team. *RStudio: Integrated Development Environment for R. Posit Software* (PBC, 2024). http://www.posit.co/

[72] Bates, D. et al. Package ‘lme4’. convergence, 12(1), 2. (2015). 10.18637/jss.v067.i01

[73] Brooks, M. E. et al. GlmmTMB balances speed and flexibility among packages for zero-inflated generalized linear mixed modeling. *R J.* **9** (2), 378–400. 10.3929/ethz-b-000240890 (2017).

[74] Dormann, C. F., Fründ, J., Blüthgen, N. & Gruber, B. Indices, graphs and null models: analyzing bipartite ecological networks. (2009).

[75] Dormann, C. F. How to be a specialist? Quantifying specialisation in pollination networks. *Netw. Biology*. **1** (1), 1–20 (2011).

[76] Wickham, H. et al. Welcome to the tidyverse. *J. open. Source Softw.* **4** (43), 1686. 10.21105/joss.01686 (2019).

[77] Wickham, H. ggplot2: Elegant Graphics for Data Analysis. Springer- New York (2016).

[78] Zuur, A. F., Ieno, E. N. & Elphick, C. S. A protocol for data exploration to avoid common statistical problems. *Methods Ecol. Evol.* **1** (1), 3–14. 10.1111/j.2041-210X.2009.00001.x (2010).

[79] Zimmermann, P., Tasser, E., Leitinger, G. & Tappeiner, U. Effects of land-use and land-cover pattern on landscape-scale biodiversity in the European alps. *Agric. Ecosyst. Environ.* **139** (1–2), 13–22. 10.1016/j.agee.2010.06.010 (2010).

[80] Kortsch, S. et al. Disentangling Temporal food web dynamics facilitates Understanding of ecosystem functioning. *J. Anim. Ecol.* **90** (5), 1205–1216. 10.1111/1365-2656.13447 (2021). 33608888 10.1111/1365-2656.13447

