# Supplementary material for: The dynamic nature of cereal food webs challenges the suitability of snapshot sampling for assessing ecosystem services
Source: Sci Rep. 2025 Nov 24;15:41614. doi: 10.1038/s41598-025-25603-2 (PMC12644813; doi:10.1038/s41598-025-25603-2)
Supplement: Supplementary file 1 — Supplementary Material 1 [file 41598_2025_25603_MOESM1_ESM.docx]

**Supplementary materials**

*Sample collection, beetle identification and regurgitation*

The beetle individuals caught in the dry pitfall traps were placed in 1.5 to 2ml tubes individually, labelled and then refrigerated in a cooling box until reaching the lab. Once there, they were taxonomically identified to the species level and made to regurgitate within their respective collection tube, after which the individual was taken from it and released outdoors. The regurgitation was induced by, heat stress from dipping the collection tubes, where the beetles were kept, into hot water for a few seconds. The regurgitate was buffered with TES buffer (0.1 M TRIS, 10 mM EDTA, 2% SDS; pH 8) and frozen at -80°C, until extraction. The contents of the wet pitfall traps were sieved through a fine mesh (0.5mm), rinsed with clean water, then put into a 100ml cup with 80% ethanol. Afterwards the contents were identified to the family level for most taxa, or species level in the case of beetles.

*Predator species wet pitfall list*

**Supplementary table 1 –** Predator counts collected from wet pitfall traps in 2020.

| Species | Fertilized | Unfertilized |
| --- | --- | --- |
| ARANEAE | 1468 | 1430 |
| STAPHYLINIDAE | 305 | 325 |
| *Bembidion properans* | 89 | 97 |
| *Poecilus cupreus* | 81 | 96 |
| *Loricera pilicornis* | 27 | 16 |
| *Clivina fossor* | 22 | 12 |
| *Pterostichus melanarius* | 18 | 36 |
| *Poecilus versicolor* | 10 | 11 |
| *Bembidion lampros* | 8 | 4 |
| *Harpalus affinis* | 8 | 19 |
| *Nebria brevicollis* | 8 | 6 |
| *Amara aenea* | 6 | 12 |
| *Agonum muelleri* | 5 | 5 |
| *Amara bifrons* | 5 | 8 |
| *Anchomenus dorsalis* | 5 | 4 |
| *Bembidion tetracolums* | 5 | 16 |
| *Bembidion quadrimaculatum* | 4 | 8 |
| *Harpalus rufipes* | 4 | 9 |
| *Amara similata* | 2 | 11 |
| *Anisodactylus binotatus* | 2 | 2 |
| *Agonum sexpunctatum* | 1 | 1 |
| *Amara familiaris* | 1 | 0 |
| *Harpalus distinguendus* | 1 | 1 |
| *Harpalus griseus* | 1 | 0 |
| *Harpalus latus* | 1 | 0 |
| *Amara montivaga* | 0 | 1 |
| *Anisodactylus signatus* | 0 | 1 |
| *Carabus granulatus* | 0 | 2 |

**Supplementary table 2** – Predator counts collected from wet pitfall traps in 2021.

| Species | Fertilized | Unfertilized |
| --- | --- | --- |
| ARANEAE | 703 | 708 |
| *Bembidion properans* | 178 | 88 |
| STAPHYLINIDAE | 169 | 199 |
| *Amara aenea* | 67 | 42 |
| *Amara similata* | 66 | 159 |
| *Poecilus cupreus* | 57 | 84 |
| *Pterostichus melanarius* | 30 | 70 |
| *Harpalus affinis* | 14 | 10 |
| *Clivina fossor* | 12 | 13 |
| *Nebria brevicollis* | 12 | 10 |
| *Poecilus versicolor* | 10 | 10 |
| *Bembidion tetracolums* | 9 | 0 |
| *Bembidion quadrimaculatum* | 6 | 2 |
| *Loricera pilicornis* | 6 | 6 |
| *Anchomenus dorsalis* | 4 | 3 |
| *Agonum sexpunctatum* | 3 | 5 |
| *Anisodactylus binotatus* | 3 | 0 |
| *Harpalus rufipes* | 3 | 4 |
| *Cylindera germanica* | 2 | 3 |
| *Amara familiaris* | 1 | 0 |
| *Bembidion lampros* | 1 | 2 |
| *Calathus melanocephalus* | 1 | 0 |
| *Harpalus caliginosus* | 1 | 0 |
| *Harpalus distinguendus* | 1 | 0 |
| *Calathus fuscipes* | 0 | 1 |

**Supplementary table 3** – Prey counts collected from wet pitfall traps in 2020 and 2021.

|  | 2020 | | 2021 | |
| --- | --- | --- | --- | --- |
| Species | Fertilized | Unfertilized | Fertilized | Unfertilized |
| ACARI | 2529 | 2276 | 363 | 423 |
| APHIDIDAE | 772 | 996 | 87 | 54 |
| COLLEMBOLA ARTHROPLEONA | 28448 | 10288 | 25753 | 14601 |
| COLLEMBOLA SYMPHYPLEONA | 3664 | 6641 | 3148 | 3785 |
| GASTROPODA | 7 | 5 | 0 | 0 |
| ISOPODA | 33 | 13 | 0 | 2 |
| LUMBRICIDAE | 2 | 14 | 0 | 0 |
| MYRIAPODA | 36 | 45 | 31 | 38 |
| *Oulema melanopus* | 11 | 7 | 9 | 7 |
| RYNCHOTA | 296 | 285 | 129 | 166 |
| THYSANOPTERA | 3 | 6 | 5 | 6 |

*Multiplex PCR assays*

- Multiplex PCR 1

The first multiplex PCR mix was comprised of 1µl PCR-grade water, 5µl KAPA2G FAST HotStart® DNA Polymerase (Roche), 1µl of the primer mix (supplementary table 1), 0.5µl of 10mg/ml BSA and 2.5µl of template DNA, for a total volume of 10µl. The thermocycling was done in a Mastercycler ® Nexus Gradient (Eppendorf, Hamburg, Germany), with the following conditions: 15 min at 95 °C, 35 cycles of 30 s at 95 °C, 90 s at 62.5 °C and 30 s at 72 °C, and 10 min at 72 °C.

**Supplementary table 4** – Primer list for multiplex PCR 1. + from Staudacher et al. (2016), * from Ye et al. (2017), ** Kuusk & Agusti (2008), *** from Rennstam Rubbmark et al. (2019).

| **Target** | **Primer code** | **Gene** | **Product (bp)** | **Final conc. in PCR (S/AS)(µM)** | **Primer sequence (5’-3’)** |
| --- | --- | --- | --- | --- | --- |
| Earthworms | S408+ | 18S | 85 | 0.8 | CGTAGTTGGATCTCGGGTCGT |
|  | A413+ |  |  |  | ATARGGGTCGGAGCTTTGTG |
| *Metopolophium* | S436* | COI | 105 | 0.4 | CCTTTATCAAATAACATTGCACATAAC |
| *dirhodum* | A440* |  |  |  | AATAAAGTTAATTGCTCCTAAAATTGAG |
| *Rhopalosiphum* | S440* | COI | 136 | 0.3 | TAATAATATAAAATTAAACCAAATTCCATTA |
| *padi* | A442* |  |  |  | TGATGTATTTAAATTACGATCAGTAAGAAG |
| *Acyrthosiphon* | S492*** | COI | 210 | 0.08 | GTCCTGATATATCATTTCCTCGC |
| *pisum* | A496*** |  |  |  | AAATTGATGAAATTCCTGCTAGG |
| Springtails | Col3F** | 18S | 231 | 0.2 | GGACGATYTTRTTRGTTCG |
|  | A415+ |  |  |  | GAATTTCACCTCTAACGTCGCAG |
| *Oulema* | Om-S2-KS-S185*** | COI | 248 | 0.2 | TTGACTTCTCCCACCTTCAA |
| *melanopus* | Om-A-KS-A184*** |  |  |  | CAAACAGAGGCATTCGATCT |
| *Sitobion avenae* | S433* | COI | 304 | 0.1 | TCATCACTTAGAATTCTTATTCGTCTT |
|  | A438* |  |  |  | AAGGTGGRTAAATAGTTCATCCTGTA |

- Multiplex PCR 2

The second PCR mix was comprised of 0.7µl PCR-grade water, 5µl of QIAGEN Multiplex PCR Master Mix (Qiagen), 1µl of the primer mix (supplementary table 2), 1µl Q-solution (Qiagen), 0.5µl of 10mg/ml BSA, 0.3µl of 30mM TMAC (Sigma-Aldrich) and 1.5µl of template DNA, for a total volume of 10µl (supplementary table 4). The thermocycling conditions were as follows: 15 min at 95 °C, 35 cycles of 30 s at 94 °C, 3 min at 62 °C and 90 s at 72 °C, and 10 min at 72 °C (supplementary table 6).

**Supplementary table 5** – Primer list for multiplex PCR 2. + primers from Sint et al. (2014), * primers from Staudacher et al. (2016).

| **Target** | **Primer codes** | **Gene** | **Product (bp)** | **Final conc. in PCR (S/AS)(µM)** | **Primer sequence (5’-3’)** |
| --- | --- | --- | --- | --- | --- |
| Hoverflies | S269+ | 18S | 86 | 0.9 | ATTAGGCTAAAACCAAGCGATTT |
|  | A270+ |  |  |  | TCGGTACAAGACCATACGATCG |
| Lacewings | S417* | 18S | 390 | 0.4 | CTGTGTCCTACACTGTTGGTTCAAT |
|  | A420* |  |  |  | AATGCCCCCATCTGTCCG |
| Spiders | S407* | 18S | ~258 | 0.3 | AATAACRATACGGGACTCTTTYGAGA |
|  | A408* |  |  |  | CGAGACAACCGGTRAAGATCAT |
| Beetles/thrips | S405* | 18S | ~208 | 0.05 | ACAGAGCTCYGACCGGAGAC |
|  | A406* |  |  |  | TTACAACCATGGTAGGCGCAG |
| Ladybeetles | S415* | 18S | 116 | 1.2 | CCCAAHTKDCCCCGC |
|  | A418* |  |  |  | GCATAAAATATTCYGGCAAAATTTC |

- Multiplex PCR 3

The first multiplex PCR mix was comprised of 1.7µl of PCR-grade water, 5µl Type-it Multiplex PCR Master Mix (Qiagen), 1µl of the primer mix (supplementary table 3), 0.5µl of 10mg/ml BSA, 0.3µl of 30mM TMAC (Sigma-Aldrich) and 1.5µl of template DNA, for a total volume of 10µl. The thermocycling conditions were as follows: 5 min at 95 °C, 35 cycles of 30 s at 95 °C, 3 min at 63 °C and 90 s at 72 °C, and 10 min at 72 °C.

**Supplementary table 6** – Primer list for multiplex PCR 3. * Primers developed for this study, all others from Staudacher et al. (2016).

| **Target** | **Primer codes** | **Gene** | **Product (bp)** | **Final conc. in PCR (S/AS)(µM)** | **Primer sequence (5’-3’)** |
| --- | --- | --- | --- | --- | --- |
| *Poecilus* | S475 | COI | 112 | 0.15/0.15 | GTGCATGATCAGGAATAGTRGGT |
|  | A486 |  |  |  | GCAGTAACAATAACATTATAAATTTGATCG |
| *Pterostichus* | S467 | 18S | 166 | 0.4/0.2 | TGATCTCGAAACGGGTCTTTTACT |
|  | A467.1 |  |  |  | CCTGTTYCATTATTCCMTGCACTA |
| *Bembidion* | S468 | 18S | ~152 | 0.1/0.1 | TGTTTAACTGGCACGTCTCGC |
|  | A470 |  |  |  | GCACCGCGACAGGATTATTG |
| *Harpalus* | S473 | COI | 349 | 0.8/0.8 | GCAGGAATAGTAGGAACTTCATTAAGC |
|  | A475 |  |  |  | AAGCTCCTCTATGWGCRATTCC |
| *Philonthus* | S927* | COI | 216 | 0.4/0.4 | TAGCTGGGATTTCCTCAATTCTC |
| *cognatus* | A927* |  |  |  | CTGTTGGGTCAAAGAATGTTGTG |
| *Philonthus* | S928* | COI | 245 | 0.2/0.2 | AGAACTAGGAAACCCCGGTACA |
| *carbonarius* | A928* |  |  |  | CGCTTTCAACTATTCTTCTCATTAAA |

*Predator species regurgitates list*

**Supplementary table 7 –** Ground beetle (Carabidae) and rove beetle (Staphylinidae) regurgitate samples collected in 2020.

| Species | Fertilized | Unfertilized |
| --- | --- | --- |
| *Poecilus cupreus* | 436 | 380 |
| *Bembidion properans* | 363 | 285 |
| *Philonthus cognatus* | 353 | 382 |
| *Philonthus carbonarius* | 104 | 74 |
| *Loricera pilicornis* | 56 | 54 |
| *Bembidion lampros* | 52 | 59 |
| *Pterostichus melanarius* | 51 | 72 |
| *Amara aenea* | 46 | 71 |
| *Bembidion tetracolum* | 26 | 37 |
| *Agonum muelleri* | 21 | 35 |
| *Bembidion quadrimaculatum* | 20 | 12 |
| *Clivina fossor* | 20 | 27 |
| *Poecilus versicolor* | 19 | 22 |
| *Anchomenus dorsalis* | 17 | 35 |
| *Harpalus affinis* | 16 | 33 |
| *Harpalus rufipes* | 15 | 13 |
| *Nebria brevicollis* | 8 | 12 |
| *Amara ingenua* | 5 | 6 |
| *Anisodactylus binotatus* | 5 | 6 |
| *Amara similata* | 4 | 18 |
| *Amara* sp. | 4 | 7 |
| *Calathus fuscipes* | 3 | 2 |
| *Agonum sexpunctatum* | 2 | 2 |
| *Carabus granulatus* | 2 | 2 |
| *Harpalus distinguendus* | 2 | 2 |
| *Amara familiaris* | 1 | 5 |
| *Amara plebeja* | 1 | 0 |
| *Bembidion* sp. | 1 | 0 |
| *Cylindera germanica* | 1 | 0 |
| *Harpalus* sp. | 1 | 0 |
| *Amara lucida* | 0 | 1 |
| *Amara lunicollis* | 0 | 1 |
| *Anisodactylus signatus* | 0 | 3 |
| *Bembidion metallina* | 0 | 1 |
| *Bembidion pilicornis* | 0 | 1 |
| *Calathus melanocephalus* | 0 | 1 |

**Supplementary table 8 –** Ground beetle (Carabidae) and rove beetle (Staphylinidae) regurgitate samples collected in 2021.

| Species | Fertilized | Unfertilized |
| --- | --- | --- |
| Philonthus cognatus | 369 | 355 |
| Poecilus cupreus | 255 | 281 |
| Bembidion properans | 229 | 155 |
| Pterostichus melanarius | 197 | 217 |
| Amara aenea | 87 | 62 |
| Philonthus carbonarius | 69 | 70 |
| Amara similata | 53 | 91 |
| Poecilus versicolor | 35 | 33 |
| Nebria brevicollis | 25 | 27 |
| Anisodactilus binotatus | 16 | 13 |
| Bembidion lampros | 16 | 11 |
| Harpalus affinis | 15 | 9 |
| Loricera pillicornis | 15 | 16 |
| Agonum muelleri | 10 | 4 |
| Clivina fossor | 10 | 18 |
| Tachyporus ruficollis | 10 | 17 |
| Harpalus rufipes | 9 | 9 |
| Agonum sexpunctatum | 7 | 1 |
| Bembidion quadrimaculatum | 7 | 0 |
| Anchomenus dorsalis | 5 | 12 |
| Bembidion tetracolum | 4 | 1 |
| Carabus granulatus | 4 | 1 |
| Amara familiaris | 3 | 0 |
| Cylindera germanica | 3 | 1 |
| Anisodactilus signatus | 1 | 3 |
| Calathus fuscipes | 1 | 3 |
| Harpalus distinguendus | 1 | 0 |
| Calathus melanocephalus | 0 | 1 |
| Tachyporus rufipes | 0 | 1 |


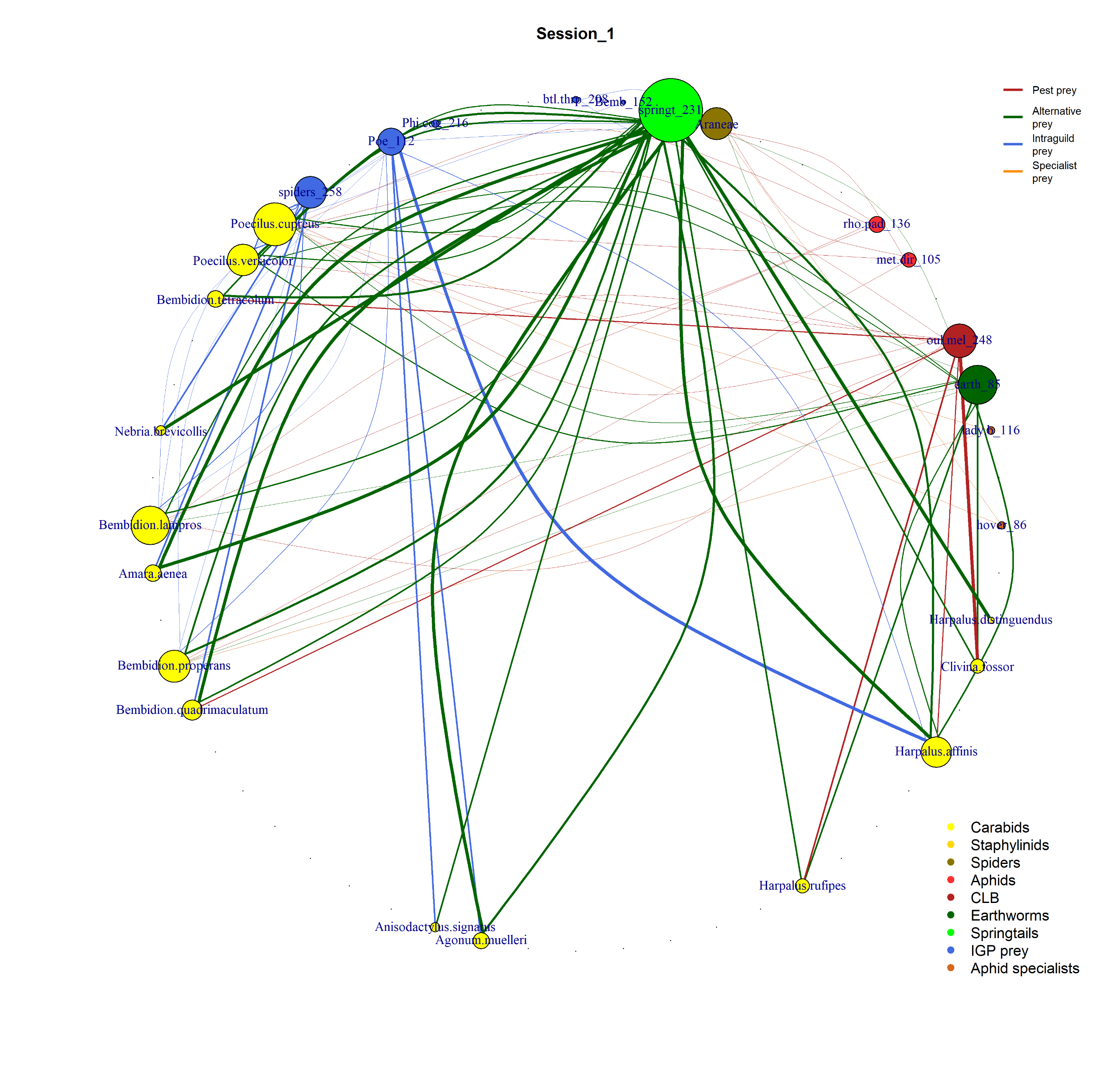


**Supplementary Figure 1** – Food web diagram for the 1st sampling session in 2020, each taxon has been colour-coded, with node diameter corresponding to the eigenvector centrality and line width representing the diet detection proportion of the trophic link.

**Predators:** yellow – Carabid beetles, orange – Staphylinid beetles, brown – spiders;

**Prey:** bright red – aphids, dark red – cereal leaf beetle, dark green – earthworms, bright green – springtails, bright blue – intraguild predation prey (beetles and spiders), dark orange – aphid specialists (hoverflies, ladybugs and lacewings).


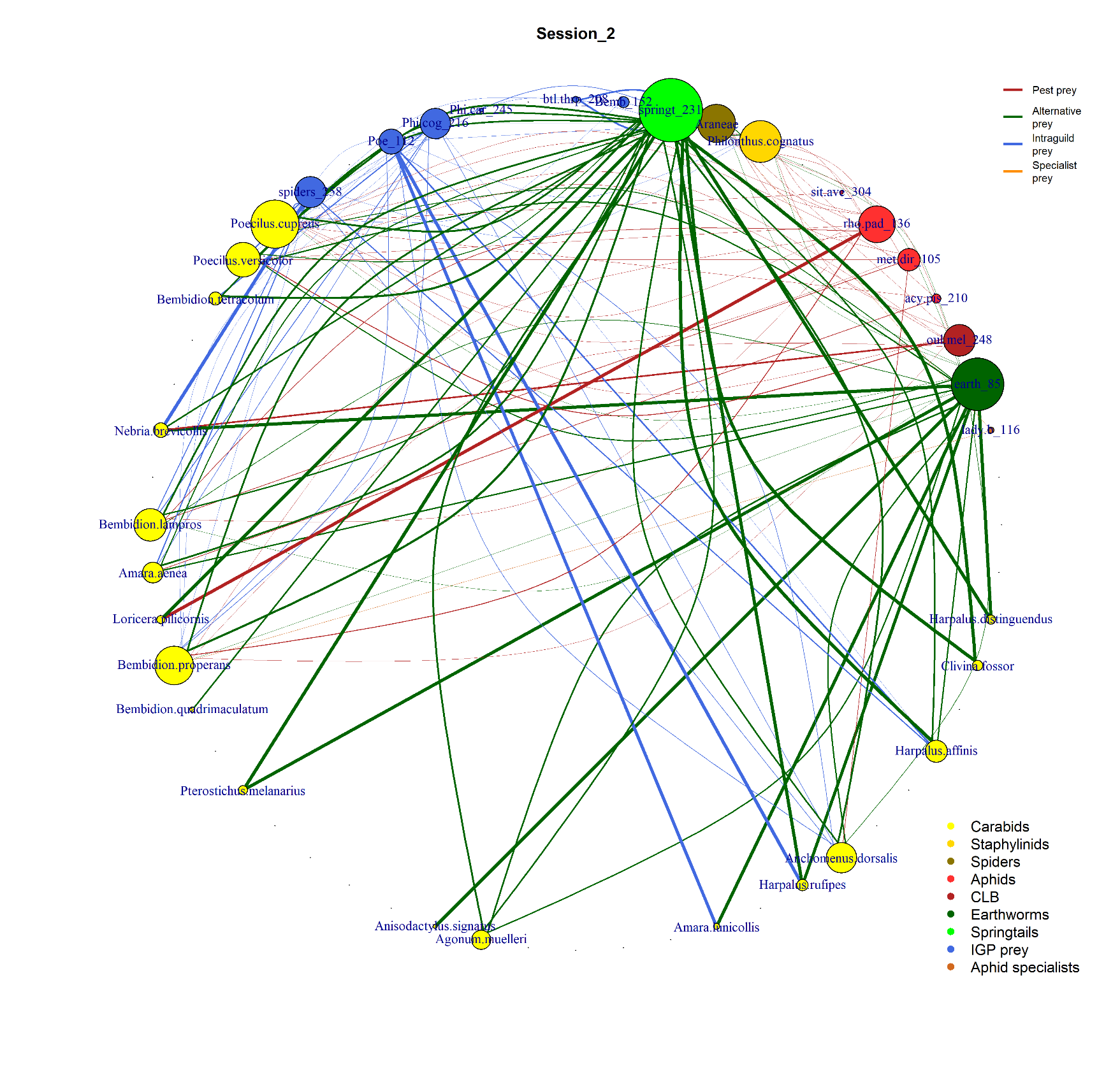


**Supplementary Figure 2** – Food web diagram for the 2nd sampling session in 2020, each taxon has been colour-coded, with node diameter corresponding to the eigenvector centrality and line width representing the diet detection proportion of the trophic link.

**Predators:** yellow – Carabid beetles, orange – Staphylinid beetles, brown – spiders;

**Prey:** bright red – aphids, dark red – cereal leaf beetle, dark green – earthworms, bright green – springtails, bright blue – intraguild predation prey (beetles and spiders), dark orange – aphid specialists (hoverflies, ladybugs and lacewings).


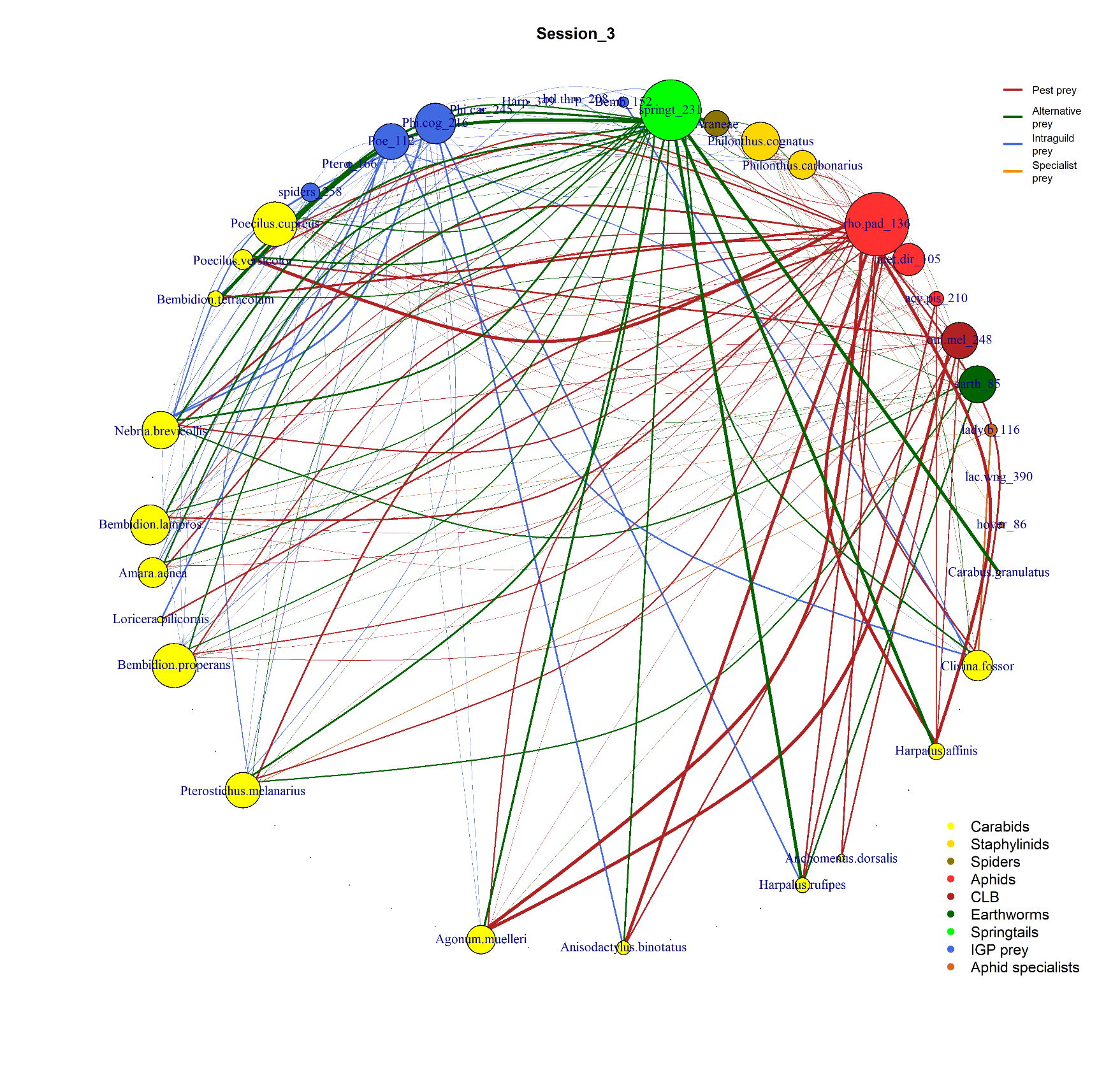


**Supplementary Figure 3** – Food web diagram for the 3rd sampling session in 2020, each taxon has been colour-coded, with node diameter corresponding to the eigenvector centrality and line width representing the diet detection proportion of the trophic link.

**Predators:** yellow – Carabid beetles, orange – Staphylinid beetles, brown – spiders;

**Prey:** bright red – aphids, dark red – cereal leaf beetle, dark green – earthworms, bright green – springtails, bright blue – intraguild predation prey (beetles and spiders), dark orange – aphid specialists (hoverflies, ladybugs and lacewings).


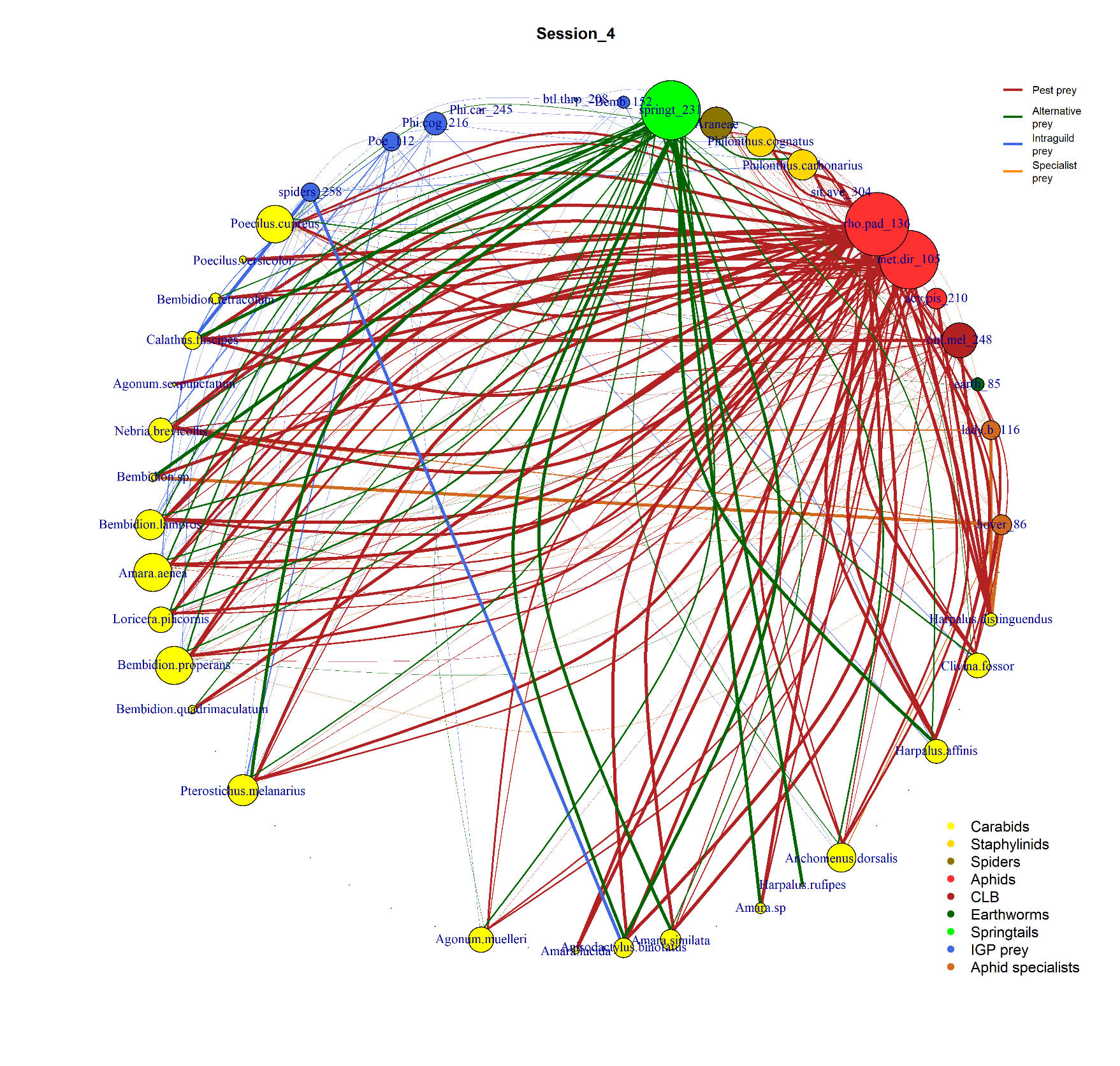


**Supplementary Figure 4** – Food web diagram for the 4th sampling session in 2020, each taxon has been colour-coded, with node diameter corresponding to the eigenvector centrality and line width representing the diet detection proportion of the trophic link.

**Predators:** yellow – Carabid beetles, orange – Staphylinid beetles, brown – spiders;

**Prey:** bright red – aphids, dark red – cereal leaf beetle, dark green – earthworms, bright green – springtails, bright blue – intraguild predation prey (beetles and spiders), dark orange – aphid specialists (hoverflies, ladybugs and lacewings).


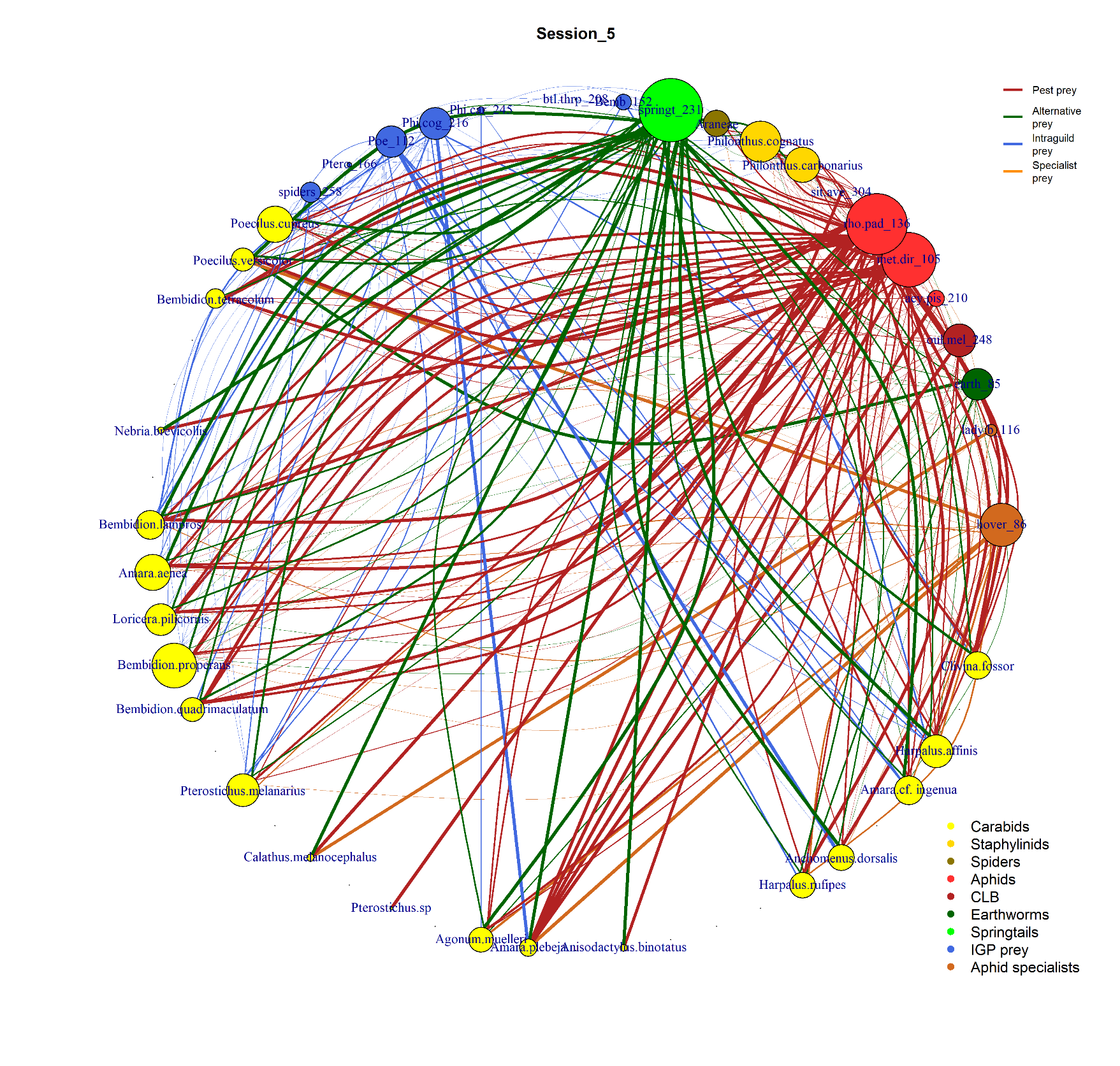


**Supplementary Figure 5** – Food web diagram for the 5th sampling session in 2020, each taxon has been colour-coded, with node diameter corresponding to the eigenvector centrality and line width representing the diet detection proportion of the trophic link.

**Predators:** yellow – Carabid beetles, orange – Staphylinid beetles, brown – spiders;

**Prey:** bright red – aphids, dark red – cereal leaf beetle, dark green – earthworms, bright green – springtails, bright blue – intraguild predation prey (beetles and spiders), dark orange – aphid specialists (hoverflies, ladybugs and lacewings).


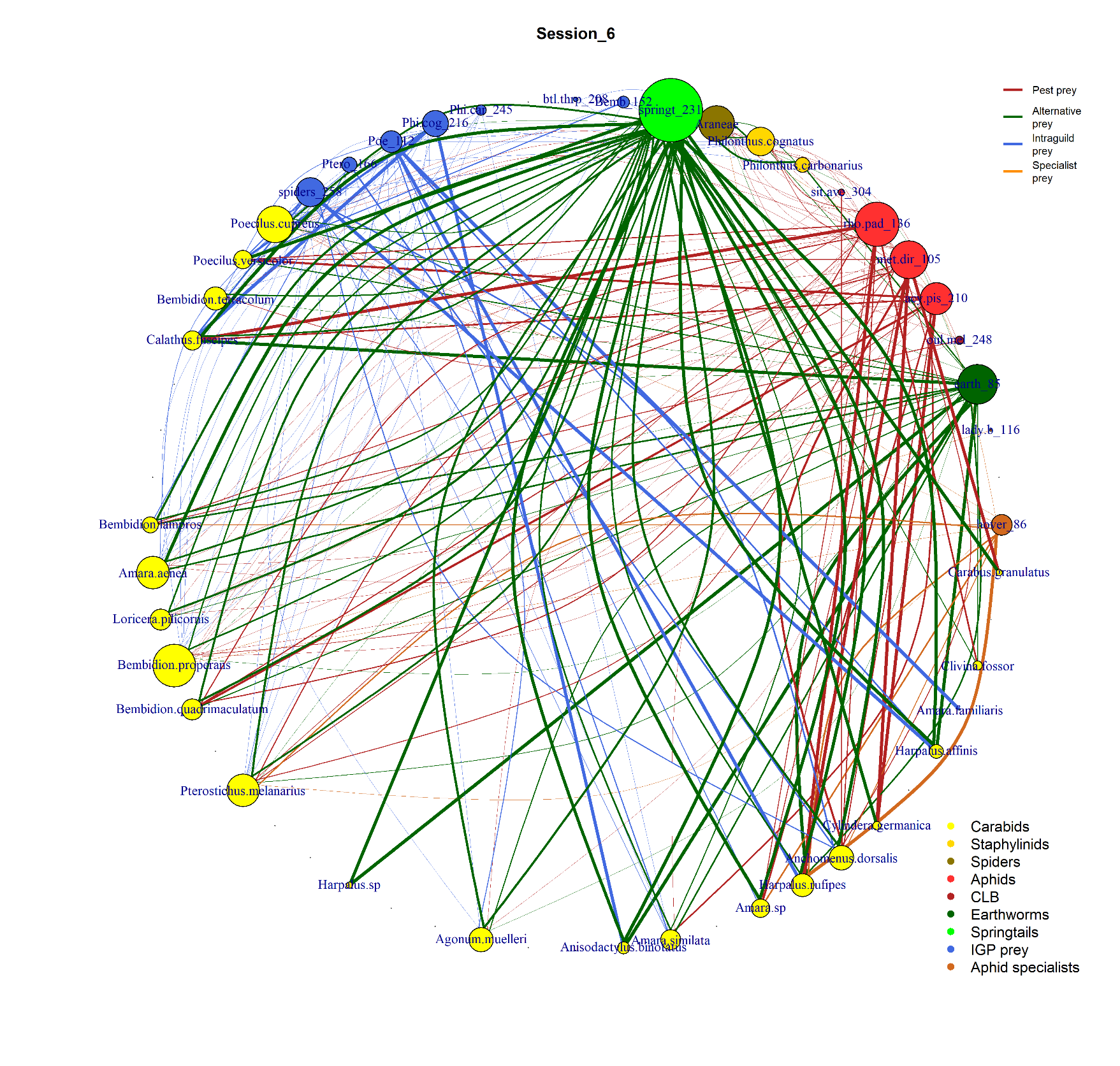


**Supplementary Figure 6** – Food web diagram for the 6th sampling session in 2020, each taxon has been colour-coded, with node diameter corresponding to the eigenvector centrality and line width representing the diet detection proportion of the trophic link.

**Predators:** yellow – Carabid beetles, orange – Staphylinid beetles, brown – spiders;

**Prey:** bright red – aphids, dark red – cereal leaf beetle, dark green – earthworms, bright green – springtails, bright blue – intraguild predation prey (beetles and spiders), dark orange – aphid specialists (hoverflies, ladybugs and lacewings).


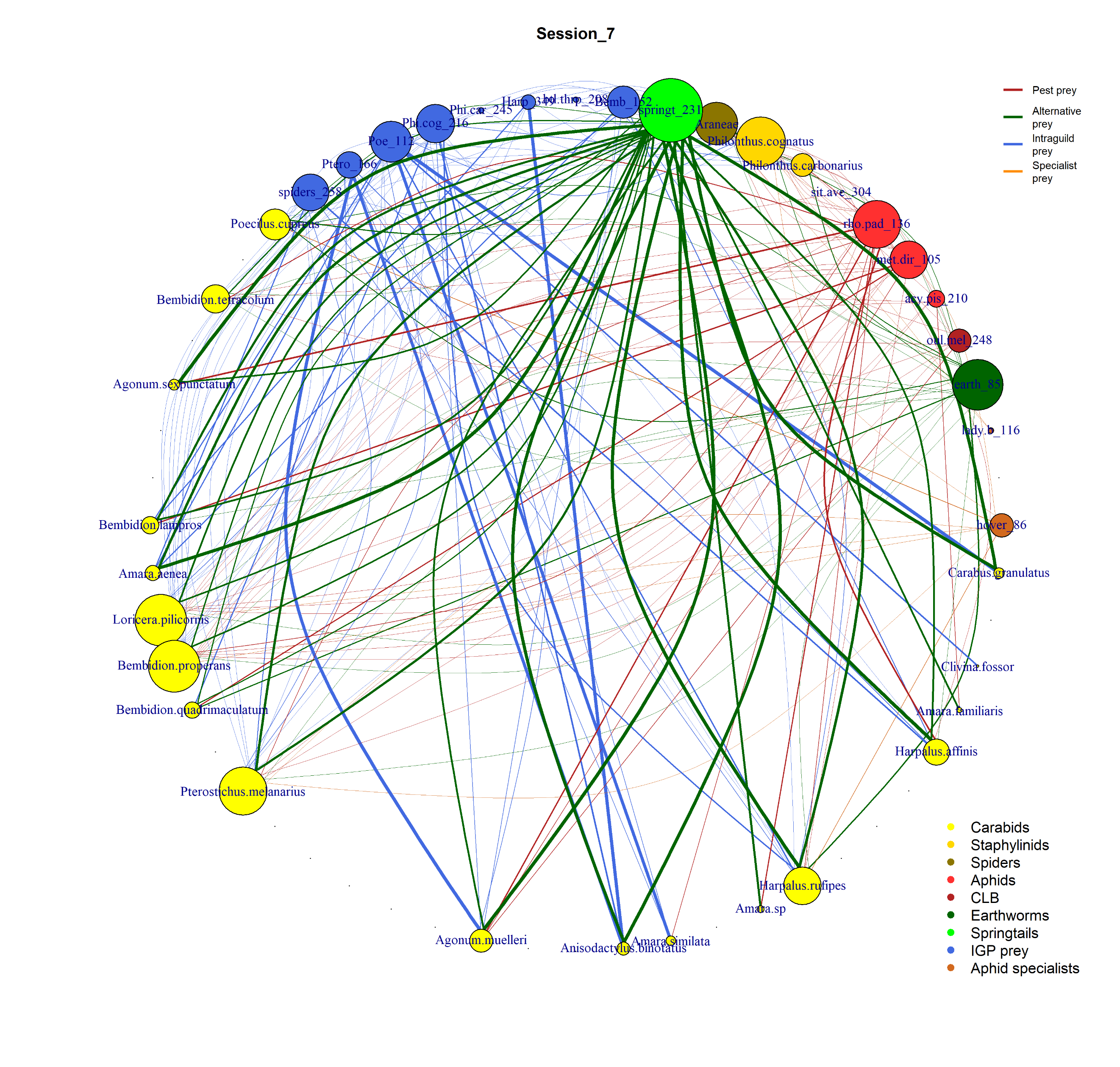


**Supplementary Figure 7** – Food web diagram for the 7th sampling session in 2020, each taxon has been colour-coded, with node diameter corresponding to the eigenvector centrality and line width representing the diet detection proportion of the trophic link.

**Predators:** yellow – Carabid beetles, orange – Staphylinid beetles, brown – spiders;

**Prey:** bright red – aphids, dark red – cereal leaf beetle, dark green – earthworms, bright green – springtails, bright blue – intraguild predation prey (beetles and spiders), dark orange – aphid specialists (hoverflies, ladybugs and lacewings).


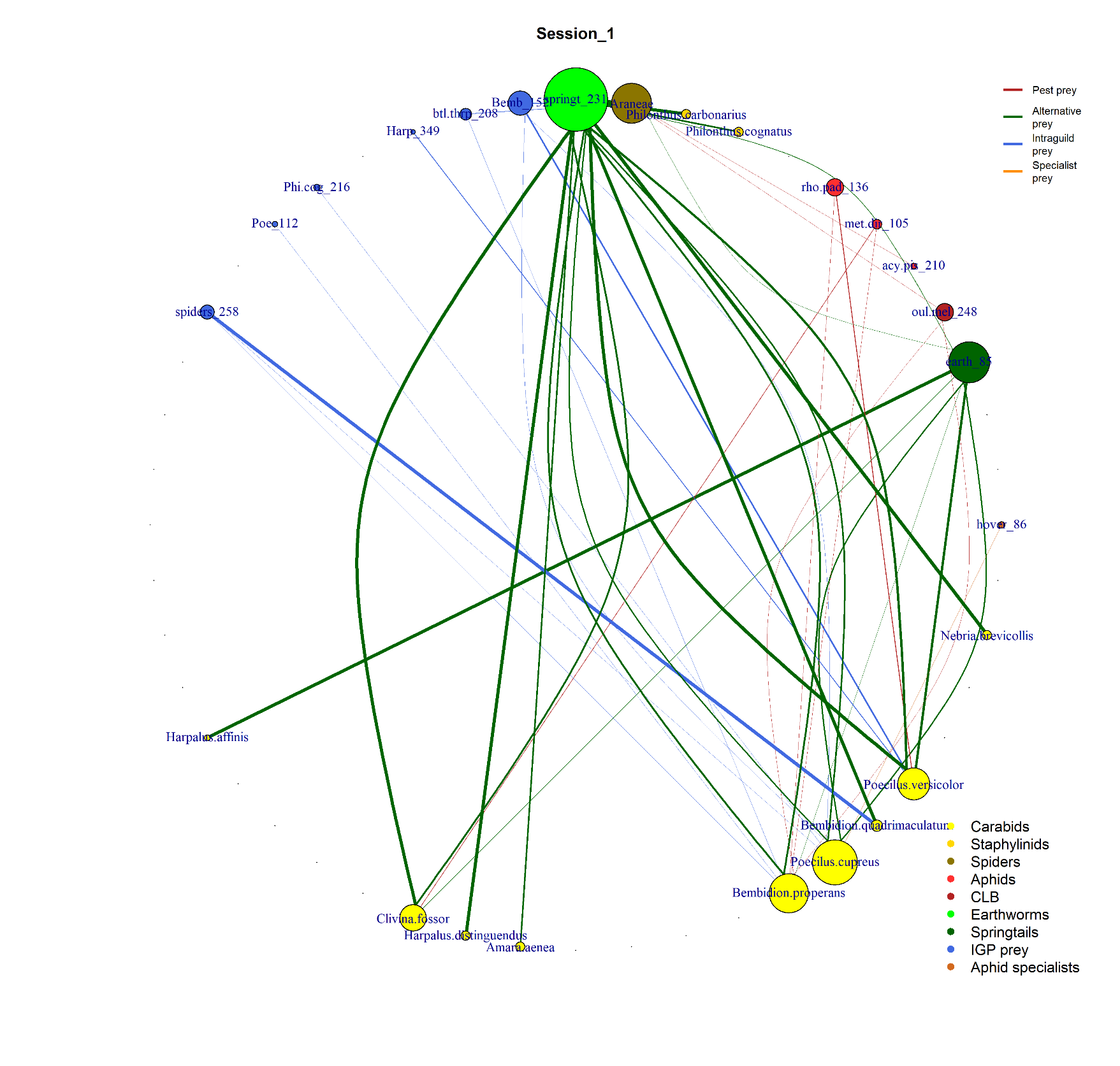


**Supplementary Figure 8** – Food web diagram for the 1st sampling session in 2021, each taxon has been colour-coded, with node diameter corresponding to the eigenvector centrality and line width representing the diet detection proportion of the trophic link.

**Predators:** yellow – Carabid beetles, orange – Staphylinid beetles, brown – spiders;

**Prey:** bright red – aphids, dark red – cereal leaf beetle, dark green – earthworms, bright green – springtails, bright blue – intraguild predation prey (beetles and spiders), dark orange – aphid specialists (hoverflies, ladybugs and lacewings).


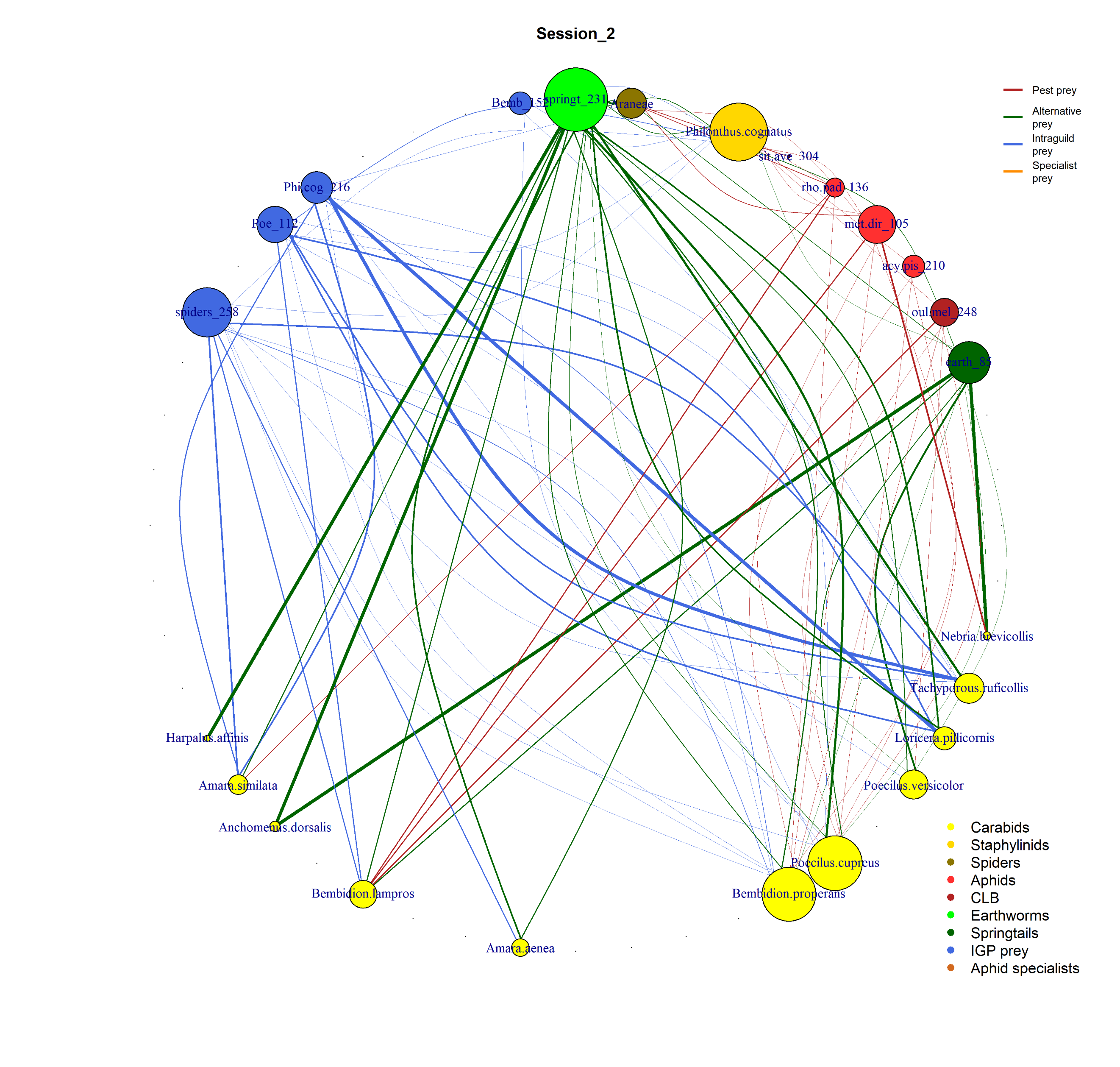


**Supplementary Figure 9** – Food web diagram for the 2nd sampling session in 2021, each taxon has been colour-coded, with node diameter corresponding to the eigenvector centrality and line width representing the diet detection proportion of the trophic link.

**Predators:** yellow – Carabid beetles, orange – Staphylinid beetles, brown – spiders;

**Prey:** bright red – aphids, dark red – cereal leaf beetle, dark green – earthworms, bright green – springtails, bright blue – intraguild predation prey (beetles and spiders), dark orange – aphid specialists (hoverflies, ladybugs and lacewings).


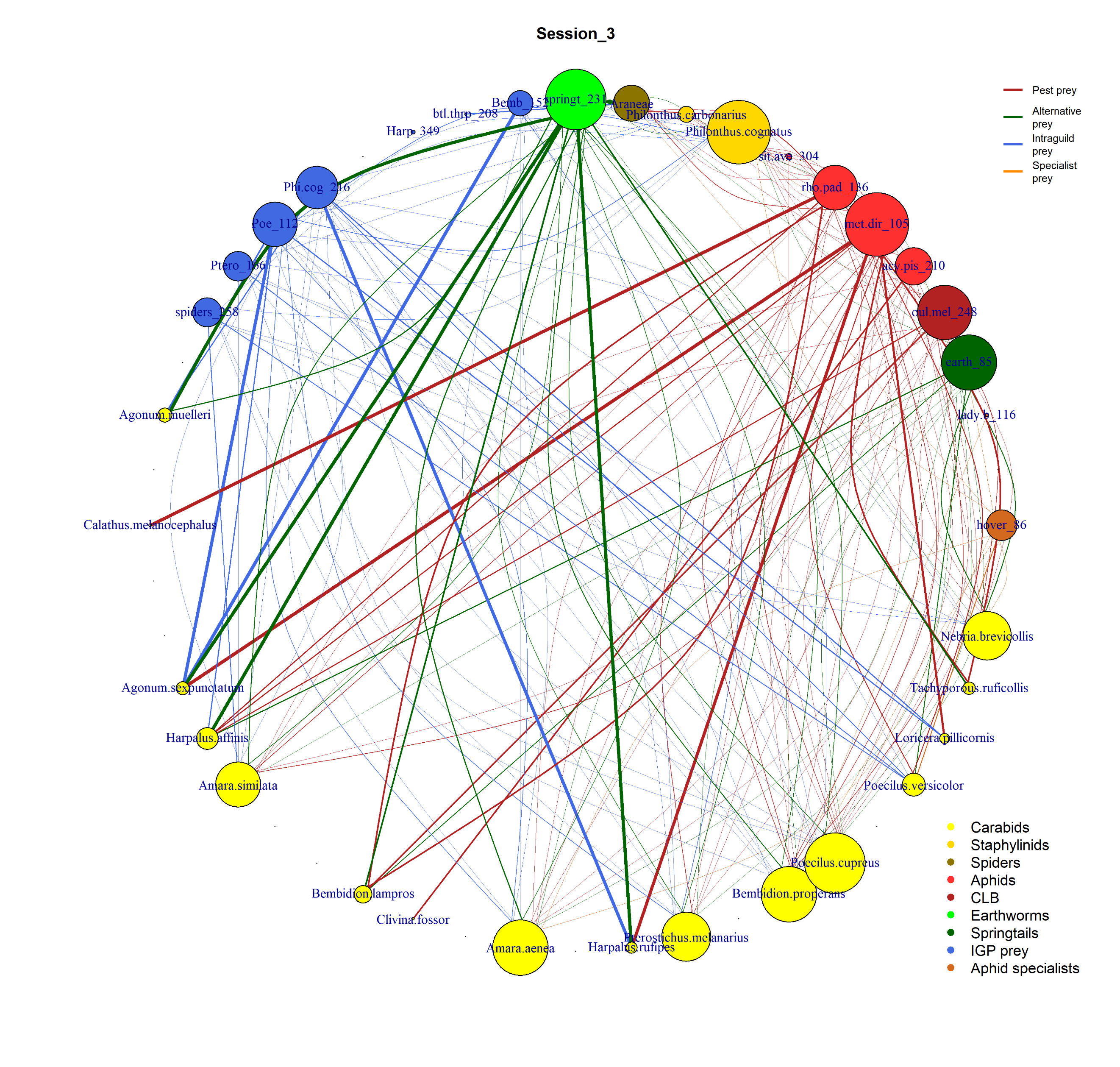


**Supplementary Figure 10** – Food web diagram for the 3rd sampling session in 2021, each taxon has been colour-coded, with node diameter corresponding to the eigenvector centrality and line width representing the diet detection proportion of the trophic link.

**Predators:** yellow – Carabid beetles, orange – Staphylinid beetles, brown – spiders;

**Prey:** bright red – aphids, dark red – cereal leaf beetle, dark green – earthworms, bright green – springtails, bright blue – intraguild predation prey (beetles and spiders), dark orange – aphid specialists (hoverflies, ladybugs and lacewings).


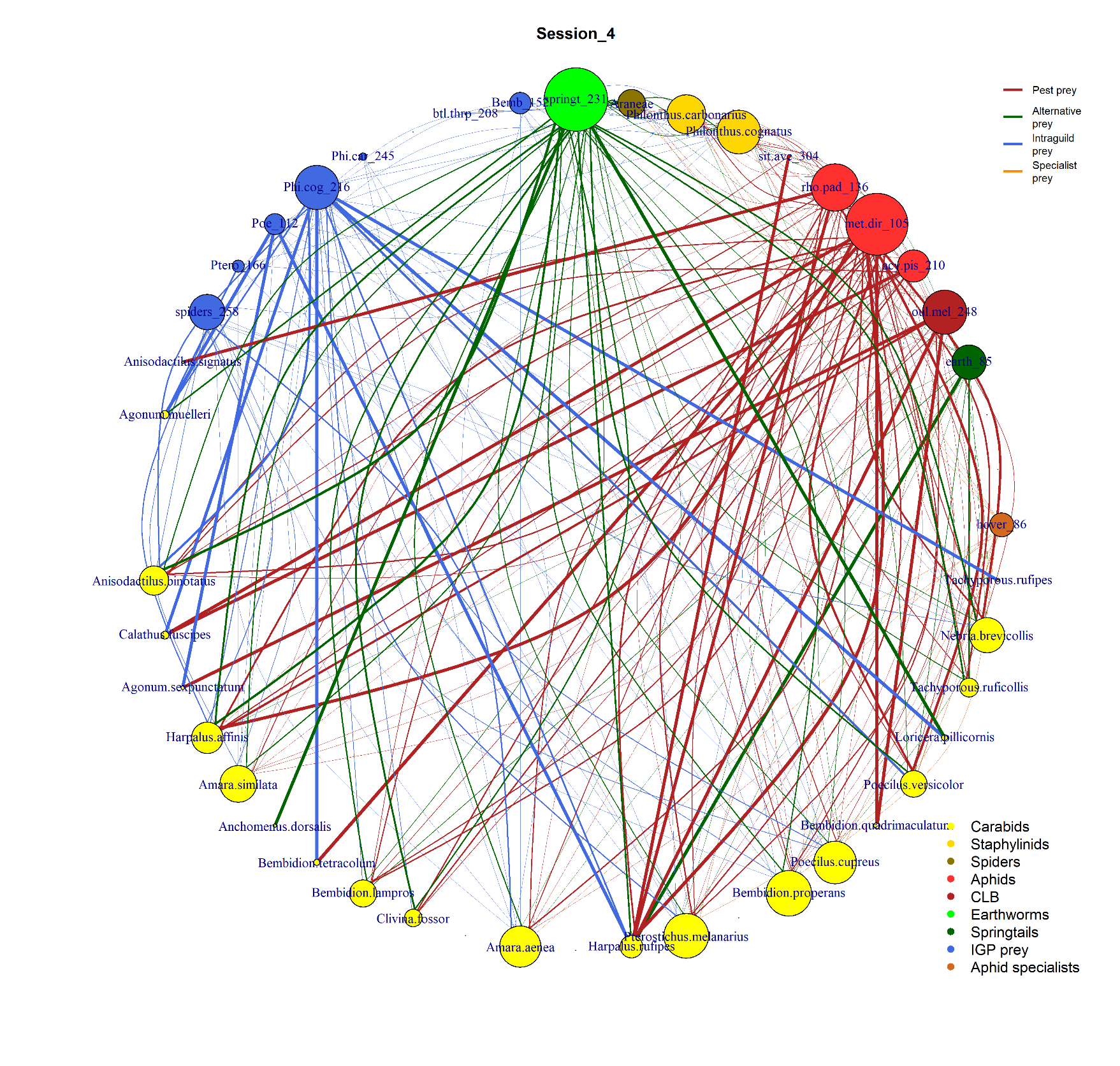


**Supplementary Figure 11** – Food web diagram for the 4th sampling session in 2021, each taxon has been colour-coded, with node diameter corresponding to the eigenvector centrality and line width representing the diet detection proportion of the trophic link.

**Predators:** yellow – Carabid beetles, orange – Staphylinid beetles, brown – spiders;

**Prey:** bright red – aphids, dark red – cereal leaf beetle, dark green – earthworms, bright green – springtails, bright blue – intraguild predation prey (beetles and spiders), dark orange – aphid specialists (hoverflies, ladybugs and lacewings).


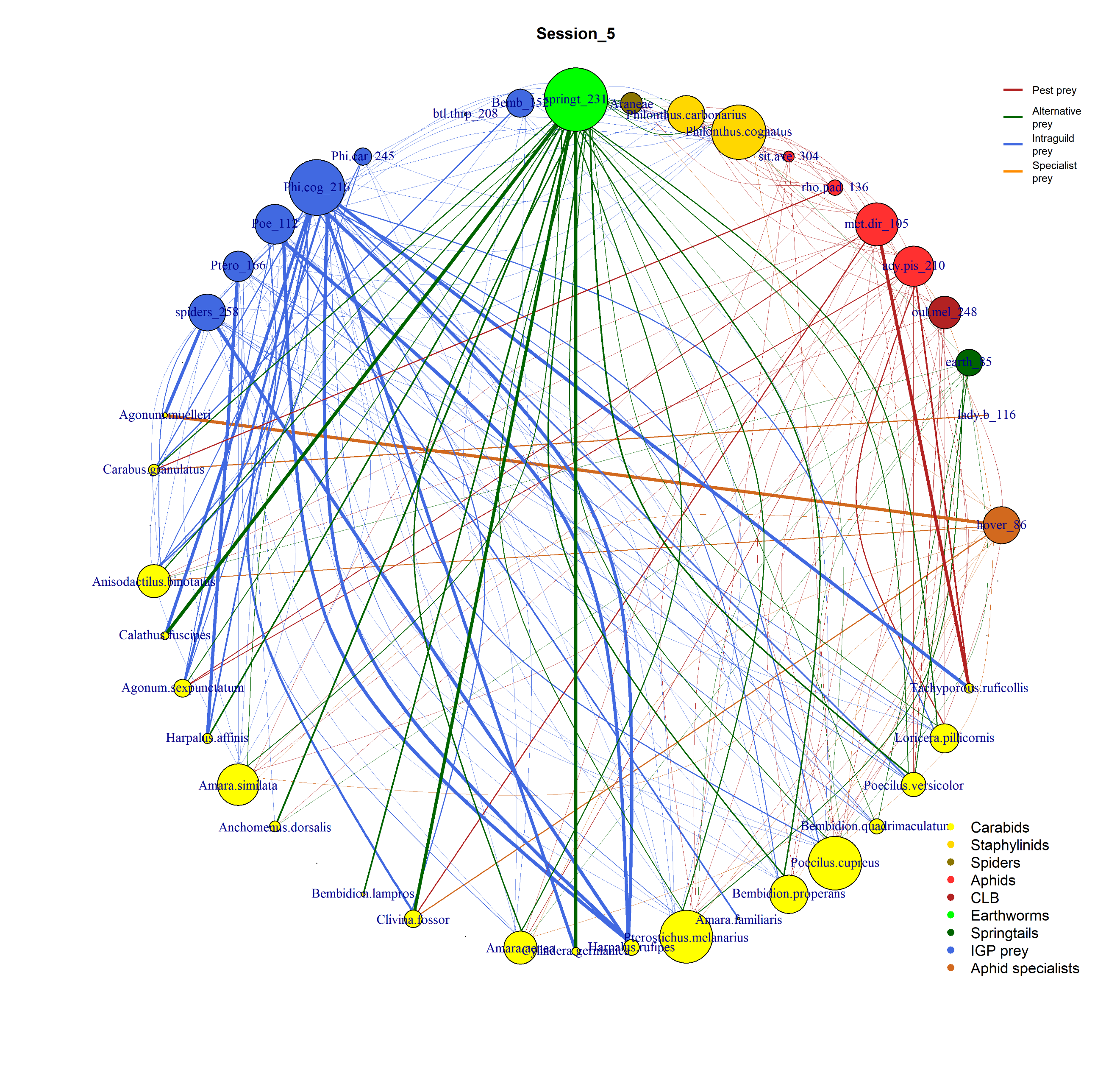


**Supplementary Figure 12** – Food web diagram for the 5th sampling session in 2021, each taxon has been colour-coded, with node diameter corresponding to the eigenvector centrality and line width representing the diet detection proportion of the trophic link.

**Predators:** yellow – Carabid beetles, orange – Staphylinid beetles, brown – spiders;

**Prey:** bright red – aphids, dark red – cereal leaf beetle, dark green – earthworms, bright green – springtails, bright blue – intraguild predation prey (beetles and spiders), dark orange – aphid specialists (hoverflies, ladybugs and lacewings).


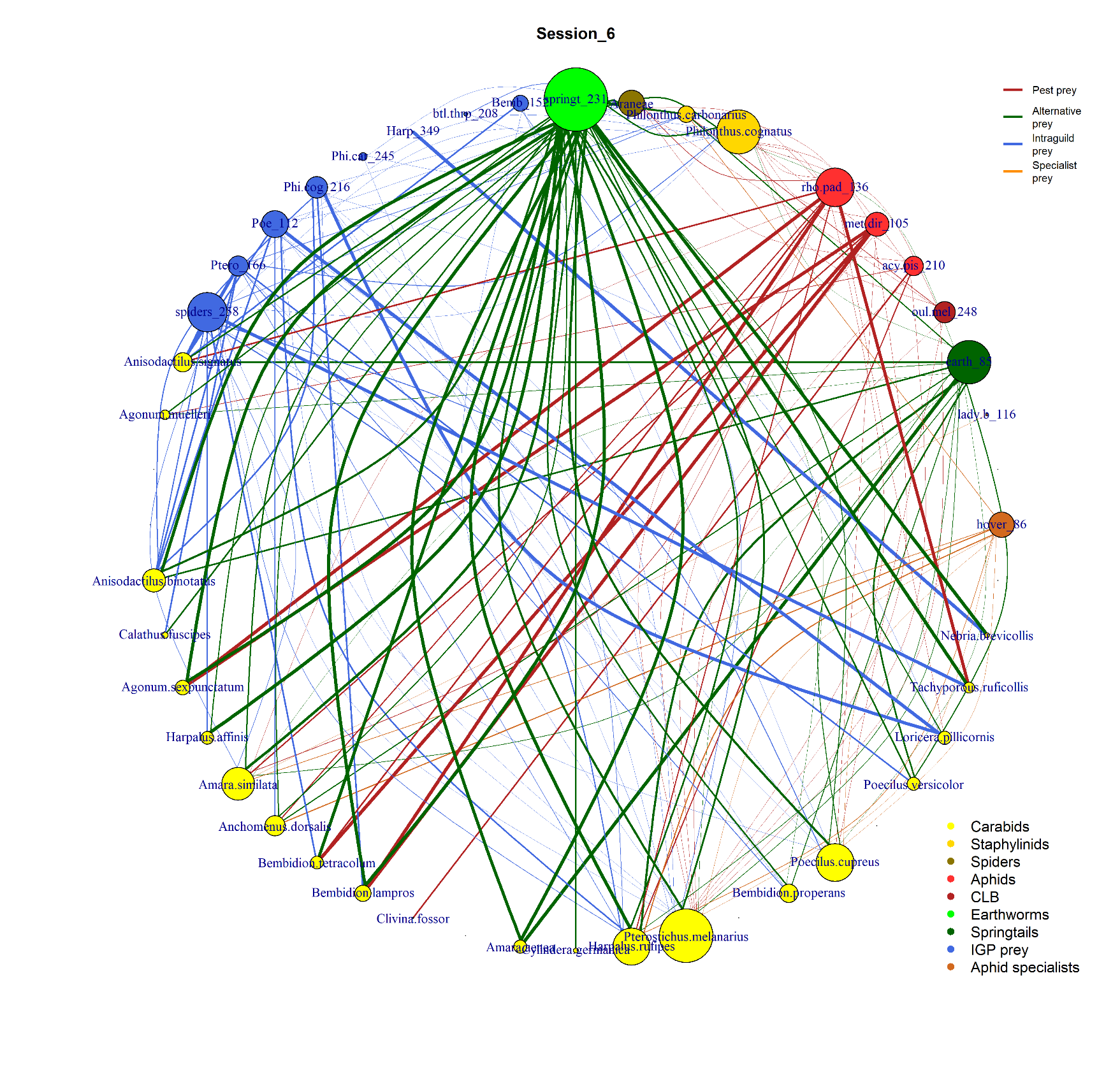


**Supplementary Figure 13** – Food web diagram for the 6th sampling session in 2021, each taxon has been colour-coded, with node diameter corresponding to the eigenvector centrality and line width representing the diet detection proportion of the trophic link.

**Predators:** yellow – Carabid beetles, orange – Staphylinid beetles, brown – spiders;

**Prey:** bright red – aphids, dark red – cereal leaf beetle, dark green – earthworms, bright green – springtails, bright blue – intraguild predation prey (beetles and spiders), dark orange – aphid specialists (hoverflies, ladybugs and lacewings).

**Supplementary Table 9** – Mean weighted connectance and link density per field and treatment in 2020.

| Session | Field | Treatment | W. connectance | Link density |
| --- | --- | --- | --- | --- |
| 1 | F1 | Fertilized | 0.3122 | 2.2845 |
| 1 | F1 | Unfertilized | 0.2879 | 2.0839 |
| 1 | F2 | Fertilized | 0.3041 | 2.3831 |
| 1 | F2 | Unfertilized | 0.3132 | 2.5957 |
| 1 | F3 | Fertilized | 0.2229 | 2.2898 |
| 1 | F3 | Unfertilized | 0.2695 | 2.1751 |
| 2 | F1 | Fertilized | 0.3269 | 1.9548 |
| 2 | F1 | Unfertilized | 0.3119 | 2.1577 |
| 2 | F2 | Fertilized | 0.2395 | 2.5017 |
| 2 | F2 | Unfertilized | 0.2011 | 2.8129 |
| 2 | F3 | Fertilized | 0.2048 | 3.4467 |
| 2 | F3 | Unfertilized | 0.2242 | 3.7275 |
| 3 | F1 | Fertilized | 0.2195 | 2.1018 |
| 3 | F1 | Unfertilized | 0.2322 | 3.2961 |
| 3 | F2 | Fertilized | 0.2659 | 2.6973 |
| 3 | F2 | Unfertilized | 0.2672 | 3.0418 |
| 3 | F3 | Fertilized | 0.2490 | 3.8629 |
| 3 | F3 | Unfertilized | 0.2315 | 4.0003 |
| 4 | F1 | Fertilized | 0.2846 | 3.4946 |
| 4 | F1 | Unfertilized | 0.2744 | 2.6912 |
| 4 | F2 | Fertilized | 0.3127 | 3.7735 |
| 4 | F2 | Unfertilized | 0.2577 | 4.3449 |
| 4 | F3 | Fertilized | 0.2993 | 4.3723 |
| 4 | F3 | Unfertilized | 0.2522 | 5.1908 |
| 5 | F1 | Fertilized | 0.3214 | 3.0471 |
| 5 | F1 | Unfertilized | 0.3256 | 3.5549 |
| 5 | F2 | Fertilized | 0.2974 | 4.4085 |
| 5 | F2 | Unfertilized | 0.2513 | 3.7959 |
| 5 | F3 | Fertilized | 0.2491 | 4.9067 |
| 5 | F3 | Unfertilized | 0.2458 | 4.2957 |
| 6 | F1 | Fertilized | 0.2228 | 2.1973 |
| 6 | F1 | Unfertilized | 0.2093 | 2.2276 |
| 6 | F2 | Fertilized | 0.2507 | 3.2972 |
| 6 | F2 | Unfertilized | 0.2399 | 3.6168 |
| 6 | F3 | Fertilized | 0.2529 | 4.1047 |
| 6 | F3 | Unfertilized | 0.2696 | 3.7408 |
| 7 | F1 | Fertilized | 0.1957 | 3.1223 |
| 7 | F1 | Unfertilized | 0.2137 | 2.9032 |
| 7 | F2 | Fertilized | 0.2009 | 3.0808 |
| 7 | F2 | Unfertilized | 0.2457 | 3.2196 |
| 7 | F3 | Fertilized | 0.2475 | 4.0255 |
| 7 | F3 | Unfertilized | 0.2636 | 3.4201 |

**Supplementary Table 10** – Mean weighted connectance and link density per field and treatment in 2021.

| Session | Field | Treatment | W. connectance | Link density |
| --- | --- | --- | --- | --- |
| 1 | F1 | Fertilized | 0.4375 | 1.0000 |
| 1 | F1 | Unfertilized | 0.3989 | 1.3932 |
| 1 | F2 | Fertilized | 0.3369 | 1.6679 |
| 1 | F2 | Unfertilized | 0.2962 | 1.0204 |
| 1 | F3 | Fertilized | 0.3125 | 1.6522 |
| 1 | F3 | Unfertilized | 0.2843 | 2.2509 |
| 2 | F1 | Fertilized | 0.2514 | 2.1847 |
| 2 | F1 | Unfertilized | 0.2703 | 2.2033 |
| 2 | F2 | Fertilized | 0.3217 | 2.1541 |
| 2 | F2 | Unfertilized | 0.3311 | 2.2031 |
| 2 | F3 | Fertilized | 0.2863 | 2.1145 |
| 2 | F3 | Unfertilized | 0.2562 | 1.9906 |
| 3 | F1 | Fertilized | 0.2519 | 2.7751 |
| 3 | F1 | Unfertilized | 0.2779 | 2.6859 |
| 3 | F2 | Fertilized | 0.1970 | 2.6659 |
| 3 | F2 | Unfertilized | 0.1735 | 2.9571 |
| 3 | F3 | Fertilized | 0.2063 | 3.5088 |
| 3 | F3 | Unfertilized | 0.2015 | 3.2612 |
| 4 | F1 | Fertilized | 0.2124 | 3.3330 |
| 4 | F1 | Unfertilized | 0.2574 | 2.6776 |
| 4 | F2 | Fertilized | 0.2186 | 3.3051 |
| 4 | F2 | Unfertilized | 0.2171 | 3.6248 |
| 4 | F3 | Fertilized | 0.2268 | 4.3318 |
| 4 | F3 | Unfertilized | 0.2629 | 3.3380 |
| 5 | F1 | Fertilized | 0.2070 | 3.8962 |
| 5 | F1 | Unfertilized | 0.1897 | 3.0705 |
| 5 | F2 | Fertilized | 0.2338 | 2.2732 |
| 5 | F2 | Unfertilized | 0.2344 | 2.7701 |
| 5 | F3 | Fertilized | 0.2459 | 2.8392 |
| 5 | F3 | Unfertilized | 0.2322 | 2.8108 |
| 6 | F1 | Fertilized | 0.2586 | 3.1189 |
| 6 | F1 | Unfertilized | 0.3184 | 1.8872 |
| 6 | F2 | Fertilized | 0.2317 | 2.6108 |
| 6 | F2 | Unfertilized | 0.2201 | 3.3540 |
| 6 | F3 | Fertilized | 0.2427 | 2.9910 |
| 6 | F3 | Unfertilized | 0.2187 | 3.2601 |


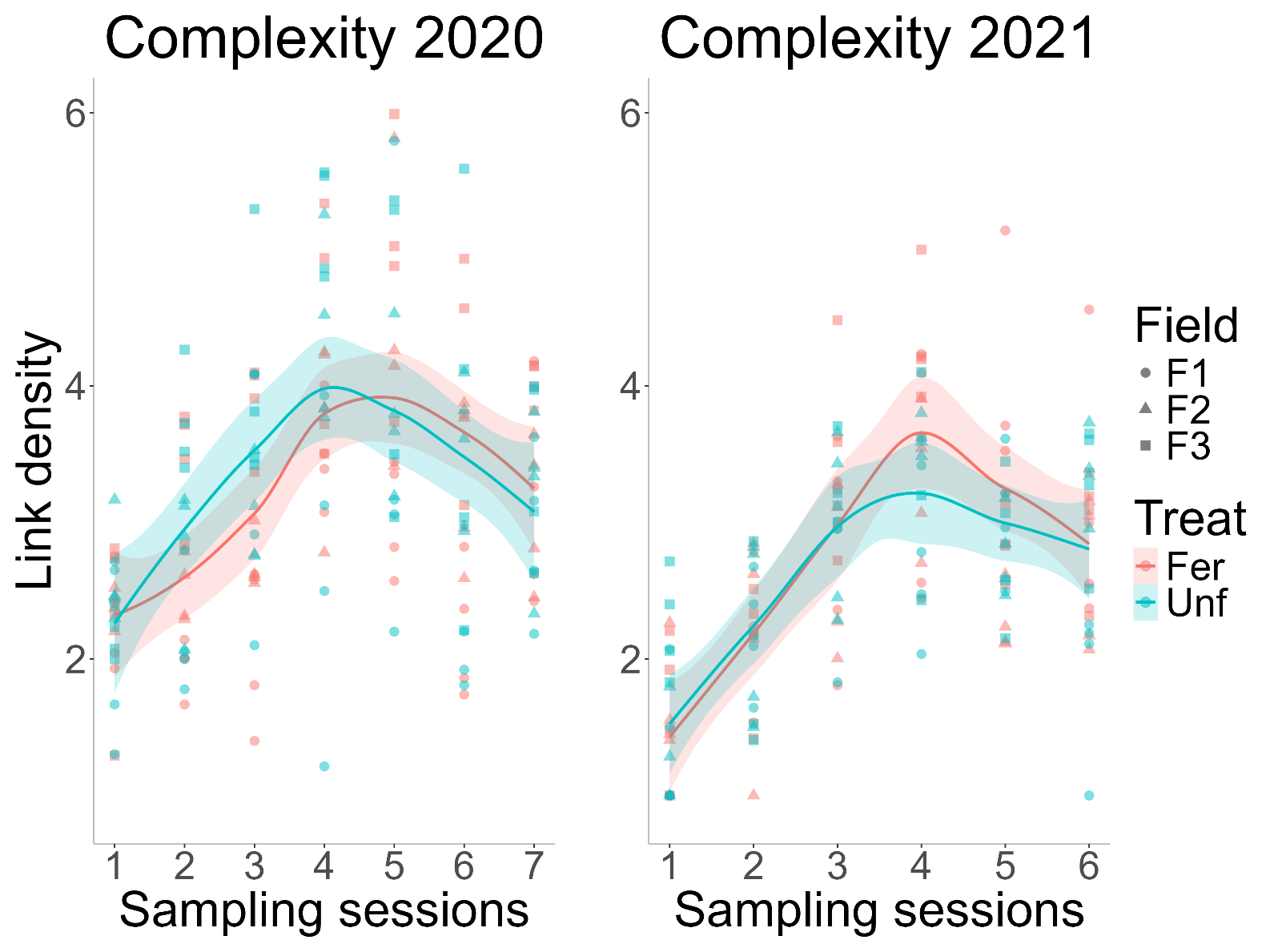


**Supplementary Figure 14** – Link density per plot and treatment in each field, across sampling sessions in 2020 and 2021.

**Supplementary Table 11** – Eigenvector centrality for predators and prey (PCR codes) taxa 2020.

| **Session** | **Taxa** | **Eigen. centrality** | **Session** | **Taxa** | **Eigen. centrality** |
| --- | --- | --- | --- | --- | --- |
| 1 | *Poecilus cupreus* | 0.6750 | 1 | oul.mel_248 (*O. melanopus)* | 0.5292 |
| 1 | *Poecilus versicolor* | 0.4964 | 1 | Phi car_245 (*P. carbonarius*) | 0.0000 |
| 1 | *Bembidion tetracolum* | 0.2641 | 1 | Phi cog_216 (*P. cognatus*) | 0.1230 |
| 1 | *Calathus fuscipes* | 0.0000 | 1 | Poe_112 (*Poecilus* genus) | 0.4267 |
| 1 | *Philonthus carbonarius* | 0.0000 | 1 | Ptero_166 (*Pterostichus* genus) | 0.0000 |
| 1 | *Philonthus cognatus* | 0.0000 | 1 | rho.pad_136 (*R. padii*) | 0.2565 |
| 1 | *Agonum sexpunctatum* | 0.0000 | 1 | sit.ave_304 (*S. avenae*) | 0.0000 |
| 1 | *Nebria brevicollis* | 0.1566 | 1 | spiders_258 (spiders) | 0.5001 |
| 1 | *Bembidion sp.* | 0.0000 | 1 | springt_231 (springtails) | 1.0000 |
| 1 | *Bembidion lampros* | 0.6036 | 2 | *Poecilus cupreus* | 0.7590 |
| 1 | *Amara aenea* | 0.2610 | 2 | *Poecilus versicolor* | 0.5469 |
| 1 | *Loricera pilicornis* | 0.0000 | 2 | *Bembidion tetracolum* | 0.2068 |
| 1 | *Bembidion properans* | 0.5061 | 2 | *Calathus fuscipes* | 0.0000 |
| 1 | *Bembidion quadrimaculatum* | 0.3163 | 2 | *Philonthus carbonarius* | 0.0000 |
| 1 | *Bembidion metallina* | 0.0000 | 2 | *Philonthus cognatus* | 0.6599 |
| 1 | *Pterostichus melanarius* | 0.0000 | 2 | *Agonum sexpunctatum* | 0.0000 |
| 1 | *Bembidion pilicornis* | 0.0000 | 2 | *Nebria brevicollis* | 0.2349 |
| 1 | Araneae | 0.5034 | 2 | *Bembidion sp.* | 0.0000 |
| 1 | *Calathus melanocephalus* | 0.0000 | 2 | *Bembidion lampros* | 0.5121 |
| 1 | *Harpalus sp.* | 0.0000 | 2 | *Amara aenea* | 0.3289 |
| 1 | *Pterostichus sp.* | 0.0000 | 2 | *Loricera pilicornis* | 0.1318 |
| 1 | *Anisodactylus signatus* | 0.1490 | 2 | *Bembidion properans* | 0.6112 |
| 1 | *Agonum muelleri* | 0.2534 | 2 | *Bembidion quadrimaculatum* | 0.0834 |
| 1 | *Amara plebeja* | 0.0000 | 2 | *Bembidion metallina* | 0.0000 |
| 1 | *Amara lucida* | 0.0000 | 2 | *Pterostichus melanarius* | 0.1523 |
| 1 | *Anisodactylus binotatus* | 0.0000 | 2 | *Bembidion pilicornis* | 0.0000 |
| 1 | *Amara similata* | 0.0000 | 2 | Araneae | 0.6003 |
| 1 | *Amara lunicollis* | 0.0000 | 2 | *Calathus melanocephalus* | 0.0000 |
| 1 | *Amara sp.* | 0.0000 | 2 | *Harpalus sp.* | 0.0000 |
| 1 | *Harpalus rufipes* | 0.2236 | 2 | *Pterostichus sp.* | 0.0000 |
| 1 | *Anchomenus dorsalis* | 0.0000 | 2 | *Anisodactylus signatus* | 0.0689 |
| 1 | *Cylindera germanica* | 0.0000 | 2 | *Agonum muelleri* | 0.3045 |
| 1 | *Amara cf. ingenua* | 0.0000 | 2 | *Amara plebeja* | 0.0000 |
| 1 | *Harpalus affinis* | 0.4811 | 2 | *Amara lucida* | 0.0000 |
| 1 | *Amara familiaris* | 0.0000 | 2 | *Anisodactylus binotatus* | 0.0000 |
| 1 | *Clivina fossor* | 0.2236 | 2 | *Amara similata* | 0.0000 |
| 1 | *Harpalus distinguendus* | 0.1044 | 2 | *Amara lunicollis* | 0.1019 |
| 1 | *Carabus granulatus* | 0.0000 | 2 | *Amara sp.* | 0.0000 |
| 1 | acy.pis_210 (*A. pisum*) | 0.0000 | 2 | *Harpalus rufipes* | 0.1852 |
| 1 | Bemb_152 (*Bembidion* genus) | 0.0705 | 2 | *Anchomenus dorsalis* | 0.4812 |
| 1 | btl.thrp_208 (beetles/thrips) | 0.1051 | 2 | *Cylindera germanica* | 0.0000 |
| 1 | earth_85 (earthworms) | 0.6128 | 2 | *Amara cf ingenua* | 0.0000 |
| 1 | Harp_349 (*Harpalus* genus) | 0.0000 | 2 | *Harpalus affinis* | 0.3497 |
| 1 | hover_86 (hoverflies) | 0.1230 | 2 | *Amara familiaris* | 0.0000 |
| 1 | lac.wng_390 (lacewings) | 0.0000 | 2 | *Clivina fossor* | 0.1667 |
| 1 | lady.b_116 (ladybugs) | 0.1233 | 2 | *Harpalus distinguendus* | 0.1523 |
| 1 | met.dir_105 (*M. dirhodum*) | 0.2284 | 2 | *Carabus granulatus* | 0.0000 |

**Supplementary Table 11** – continued.

| Session | Taxa | Eigen. centrality | Session | Taxa | Eigen. centrality |
| --- | --- | --- | --- | --- | --- |
| 2 | acy.pis_210 (*A. pisum*) | 0.1457 | 3 | *Harpalus rufipes* | 0.2398 |
| 2 | Bemb_152 (*Bembidion* genus) | 0.1816 | 3 | *Anchomenus dorsalis* | 0.1107 |
| 2 | btl.thrp_208 (beetles/thrips) | 0.1001 | 3 | *Cylindera germanica* | 0.0000 |
| 2 | earth_85 (earthworms) | 0.8265 | 3 | *Amara cf ingenua* | 0.0000 |
| 2 | Harp_349 (*Harpalus* genus) | 0.0000 | 3 | *Harpalus affinis* | 0.2645 |
| 2 | hover_86 (hoverflies) | 0.0000 | 3 | *Amara familiaris* | 0.0000 |
| 2 | lac.wng_390 (lacewings) | 0.0000 | 3 | *Clivina fossor* | 0.4830 |
| 2 | lady.b_116 (ladybugs) | 0.1010 | 3 | *Harpalus distinguendus* | 0.0000 |
| 2 | met.dir_105 (*M. dirhodum*) | 0.3551 | 3 | *Carabus granulatus* | 0.0673 |
| 2 | oul.mel_248 (*O. melanopus)* | 0.4993 | 3 | acy.pis_210 (*A. pisum*) | 0.2279 |
| 2 | Phi car_245 (*P. carbonarius*) | 0.0633 | 3 | Bemb_152 (*Bembidion* genus) | 0.1661 |
| 2 | Phi cog_216 (*P. cognatus*) | 0.4808 | 3 | btl.thrp_208 (beetles/thrips) | 0.0577 |
| 2 | Poe_112 (*Poecilus* genus) | 0.3953 | 3 | earth_85 (earthworms) | 0.5827 |
| 2 | Ptero_166 (*Pterostichus* genus) | 0.0000 | 3 | Harp_349 (*Harpalus* genus) | 0.0417 |
| 2 | rho.pad_136 (*R. padii*) | 0.5806 | 3 | hover_86 (hoverflies) | 0.0924 |
| 2 | sit.ave_304 (*S. avenae*) | 0.0633 | 3 | lac.wng_390 (lacewings) | 0.0429 |
| 2 | spiders_258 (spiders) | 0.4921 | 3 | lady.b_116 (ladybugs) | 0.1992 |
| 2 | springt_231 (springtails) | 1.0000 | 3 | met.dir_105 (*M. dirhodum*) | 0.5096 |
| 3 | *Poecilus cupreus* | 0.7033 | 3 | oul.mel_248 (*O. melanopus)* | 0.5730 |
| 3 | *Poecilus versicolor* | 0.3157 | 3 | Phi car_245 (*P. carbonarius*) | 0.0429 |
| 3 | *Bembidion tetracolum* | 0.2451 | 3 | Phi cog_216 (*P. cognatus*) | 0.6400 |
| 3 | *Calathus fuscipes* | 0.0000 | 3 | Poe_112 (*Poecilus* genus) | 0.5696 |
| 3 | *Philonthus carbonarius* | 0.4559 | 3 | Ptero_166 (*Pterostichus* genus) | 0.0924 |
| 3 | *Philonthus cognatus* | 0.6091 | 3 | rho.pad_136 (*R. padii*) | 1.0000 |
| 3 | *Agonum sexpunctatum* | 0.0000 | 3 | sit.ave_304 (*S. avenae*) | 0.0000 |
| 3 | *Nebria brevicollis* | 0.5918 | 3 | spiders_258 (spiders) | 0.2957 |
| 3 | *Bembidion sp.* | 0.0000 | 3 | springt_231 (springtails) | 0.9561 |
| 3 | *Bembidion lampros* | 0.6291 | 4 | *Poecilus cupreus* | 0.5945 |
| 3 | *Amara aenea* | 0.4699 | 4 | *Poecilus versicolor* | 0.1179 |
| 3 | *Loricera pilicornis* | 0.1105 | 4 | *Bembidion tetracolum* | 0.1750 |
| 3 | *Bembidion properans* | 0.6968 | 4 | *Calathus fuscipes* | 0.2881 |
| 3 | *Bembidion quadrimaculatum* | 0.0000 | 4 | *Philonthus carbonarius* | 0.4811 |
| 3 | *Bembidion metallina* | 0.0000 | 4 | *Philonthus cognatus* | 0.4725 |
| 3 | *Pterostichus melanarius* | 0.5583 | 4 | *Agonum sexpunctatum* | 0.0613 |
| 3 | *Bembidion pilicornis* | 0.0000 | 4 | *Nebria brevicollis* | 0.3828 |
| 3 | Araneae | 0.4097 | 4 | *Bembidion sp.* | 0.1379 |
| 3 | *Calathus melanocephalus* | 0.0000 | 4 | *Bembidion lampros* | 0.4763 |
| 3 | *Harpalus sp.* | 0.0000 | 4 | *Amara aenea* | 0.5997 |
| 3 | *Pterostichus sp.* | 0.0000 | 4 | *Loricera pilicornis* | 0.4040 |
| 3 | *Anisodactylus signatus* | 0.0000 | 4 | *Bembidion properans* | 0.6016 |
| 3 | *Agonum muelleri* | 0.4508 | 4 | *Bembidion quadrimaculatum* | 0.1386 |
| 3 | *Amara plebeja* | 0.0000 | 4 | *Bembidion metallina* | 0.0000 |
| 3 | *Amara lucida* | 0.0000 | 4 | *Pterostichus melanarius* | 0.4930 |
| 3 | *Anisodactylus binotatus* | 0.2231 | 4 | *Bembidion pilicornis* | 0.0000 |
| 3 | *Amara similata* | 0.0000 | 4 | Araneae | 0.5190 |
| 3 | *Amara lunicollis* | 0.0000 | 4 | *Calathus melanocephalus* | 0.0000 |
| 3 | *Amara sp.* | 0.0000 | 4 | *Harpalus sp.* | 0.0000 |

**Supplementary Table 11** – continued.

| Session | Taxa | Eigen. centrality | Session | Taxa | Eigen. centrality |
| --- | --- | --- | --- | --- | --- |
| 4 | *Pterostichus sp.* | 0.0000 | 5 | *Loricera pilicornis* | 0.5021 |
| 4 | *Anisodactylus signatus* | 0.0000 | 5 | *Bembidion properans* | 0.7061 |
| 4 | *Agonum muelleri* | 0.4021 | 5 | *Bembidion quadrimaculatum* | 0.3795 |
| 4 | *Amara plebeja* | 0.0000 | 5 | *Bembidion metallina* | 0.0000 |
| 4 | *Amara lucida* | 0.1179 | 5 | *Pterostichus melanarius* | 0.5199 |
| 4 | *Anisodactylus binotatus* | 0.3114 | 5 | *Bembidion pilicornis* | 0.0000 |
| 4 | *Amara similata* | 0.3272 | 5 | Araneae | 0.4182 |
| 4 | *Amara lunicollis* | 0.0000 | 5 | *Calathus melanocephalus* | 0.1225 |
| 4 | *Amara sp.* | 0.1750 | 5 | *Harpalus sp.* | 0.0000 |
| 4 | *Harpalus rufipes* | 0.0571 | 5 | *Pterostichus sp.* | 0.0548 |
| 4 | *Anchomenus dorsalis* | 0.4597 | 5 | *Anisodactylus signatus* | 0.0000 |
| 4 | *Cylindera germanica* | 0.0000 | 5 | *Agonum muelleri* | 0.3946 |
| 4 | *Amara cf ingenua* | 0.0000 | 5 | *Amara plebeja* | 0.2746 |
| 4 | *Harpalus affinis* | 0.3803 | 5 | *Amara lucida* | 0.0000 |
| 4 | *Amara familiaris* | 0.0000 | 5 | *Anisodactylus binotatus* | 0.1118 |
| 4 | *Clivina fossor* | 0.3926 | 5 | *Amara similata* | 0.0000 |
| 4 | *Harpalus distinguendus* | 0.2097 | 5 | *Amara lunicollis* | 0.0000 |
| 4 | *Carabus granulatus* | 0.0000 | 5 | *Amara sp.* | 0.0000 |
| 4 | acy.pis_210 (*A. pisum*) | 0.3291 | 5 | *Harpalus rufipes* | 0.4066 |
| 4 | Bemb_152 (*Bembidion* genus) | 0.1951 | 5 | *Anchomenus dorsalis* | 0.4099 |
| 4 | btl.thrp_208 (beetles/thrips) | 0.0637 | 5 | *Cylindera germanica* | 0.0000 |
| 4 | earth_85 (earthworms) | 0.2078 | 5 | *Amara cf ingenua* | 0.4548 |
| 4 | Harp_349 (*Harpalus* genus) | 0.0000 | 5 | *Harpalus affinis* | 0.5197 |
| 4 | hover_86 (hoverflies) | 0.3173 | 5 | *Amara familiaris* | 0.0000 |
| 4 | lac.wng_390 (lacewings) | 0.0000 | 5 | *Clivina fossor* | 0.4333 |
| 4 | lady.b_116 (ladybugs) | 0.2994 | 5 | *Harpalus distinguendus* | 0.0000 |
| 4 | met.dir_105 (*M. dirhodum*) | 0.9224 | 5 | *Carabus granulatus* | 0.0000 |
| 4 | oul.mel_248 (*O. melanopus)* | 0.5510 | 5 | acy.pis_210 (*A. pisum*) | 0.2584 |
| 4 | Phi car_245 (*P. carbonarius*) | 0.0318 | 5 | Bemb_152 (*Bembidion* genus) | 0.2490 |
| 4 | Phi cog_216 (*P. cognatus*) | 0.3651 | 5 | btl.thrp_208 (beetles/thrips) | 0.0238 |
| 4 | Poe_112 (*Poecilus* genus) | 0.2985 | 5 | earth_85 (earthworms) | 0.5007 |
| 4 | Ptero_166 (*Pterostichus* genus) | 0.0000 | 5 | Harp_349 (*Harpalus* genus) | 0.0000 |
| 4 | rho.pad_136 (*R. padii*) | 1.0000 | 5 | hover_86 (hoverflies) | 0.6792 |
| 4 | sit.ave_304 (*S. avenae*) | 0.0318 | 5 | lac.wng_390 (lacewings) | 0.0000 |
| 4 | spiders_258 (spiders) | 0.2930 | 5 | lady.b_116 (ladybugs) | 0.1867 |
| 4 | springt_231 (springtails) | 0.9313 | 5 | met.dir_105 (*M. dirhodum*) | 0.8548 |
| 5 | *Poecilus cupreus* | 0.5723 | 5 | oul.mel_248 (*O. melanopus)* | 0.5199 |
| 5 | *Poecilus versicolor* | 0.3682 | 5 | Phi car_245 (*P. carbonarius*) | 0.1086 |
| 5 | *Bembidion tetracolum* | 0.3085 | 5 | Phi cog_216 (*P. cognatus*) | 0.5036 |
| 5 | *Calathus fuscipes* | 0.0000 | 5 | Poe_112 (*Poecilus* genus) | 0.4951 |
| 5 | *Philonthus carbonarius* | 0.5566 | 5 | Ptero_166 (*Pterostichus* genus) | 0.0772 |
| 5 | *Philonthus cognatus* | 0.6480 | 5 | rho.pad_136 (*R. padii*) | 0.9617 |
| 5 | *Agonum sexpunctatum* | 0.0000 | 5 | sit.ave_304 (*S. avenae*) | 0.0395 |
| 5 | *Nebria brevicollis* | 0.1057 | 5 | spiders_258 (spiders) | 0.3235 |
| 5 | *Bembidion sp.* | 0.0000 | 5 | springt_231 (springtails) | 1.0000 |
| 5 | *Bembidion lampros* | 0.4539 | 6 | *Poecilus cupreus* | 0.5792 |
| 5 | *Amara aenea* | 0.5660 | 6 | *Poecilus versicolor* | 0.2923 |

**Supplementary Table 11** – continued.

| Session | Taxa | Eigen. centrality | Session | Taxa | Eigen. centrality |
| --- | --- | --- | --- | --- | --- |
| 6 | *Bembidion tetracolum* | 0.3679 | 6 | Phi cog_216 (*P. cognatus*) | 0.4148 |
| 6 | *Calathus fuscipes* | 0.3058 | 6 | Poe_112 (*Poecilus* genus) | 0.3483 |
| 6 | *Philonthus carbonarius* | 0.2368 | 6 | Ptero_166 (*Pterostichus* genus) | 0.2402 |
| 6 | *Philonthus cognatus* | 0.4528 | 6 | rho.pad_136 (*R. padii*) | 0.6965 |
| 6 | *Agonum sexpunctatum* | 0.0000 | 6 | sit.ave_304 (*S. avenae*) | 0.1057 |
| 6 | *Nebria brevicollis* | 0.0000 | 6 | spiders_258 (spiders) | 0.4576 |
| 6 | *Bembidion sp.* | 0.0000 | 6 | springt_231 (springtails) | 1.0000 |
| 6 | *Bembidion lampros* | 0.2556 | 7 | *Poecilus cupreus* | 0.4904 |
| 6 | *Amara aenea* | 0.5148 | 7 | *Poecilus versicolor* | 0.0000 |
| 6 | *Loricera pilicornis* | 0.3333 | 7 | *Bembidion tetracolum* | 0.4480 |
| 6 | *Bembidion properans* | 0.6712 | 7 | *Calathus fuscipes* | 0.0000 |
| 6 | *Bembidion quadrimaculatum* | 0.3333 | 7 | *Philonthus carbonarius* | 0.3618 |
| 6 | *Bembidion metallina* | 0.0000 | 7 | *Philonthus cognatus* | 0.7813 |
| 6 | *Pterostichus melanarius* | 0.5136 | 7 | *Agonum sexpunctatum* | 0.1774 |
| 6 | *Bembidion pilicornis* | 0.0000 | 7 | *Nebria brevicollis* | 0.0000 |
| 6 | Araneae | 0.5645 | 7 | *Bembidion sp.* | 0.0000 |
| 6 | *Calathus melanocephalus* | 0.0000 | 7 | *Bembidion lampros* | 0.2753 |
| 6 | *Harpalus sp.* | 0.1059 | 7 | *Amara aenea* | 0.2408 |
| 6 | *Pterostichus sp.* | 0.0000 | 7 | *Loricera pilicornis* | 0.8102 |
| 6 | *Anisodactylus signatus* | 0.0000 | 7 | *Bembidion properans* | 0.8159 |
| 6 | *Agonum muelleri* | 0.3845 | 7 | *Bembidion quadrimaculatum* | 0.2578 |
| 6 | *Amara plebeja* | 0.0000 | 7 | *Bembidion metallina* | 0.0000 |
| 6 | *Amara lucida* | 0.0000 | 7 | *Pterostichus melanarius* | 0.7556 |
| 6 | *Anisodactylus binotatus* | 0.1979 | 7 | *Bembidion pilicornis* | 0.0000 |
| 6 | *Amara similata* | 0.3157 | 7 | Araneae | 0.6688 |
| 6 | *Amara lunicollis* | 0.0000 | 7 | *Calathus melanocephalus* | 0.0000 |
| 6 | *Amara sp.* | 0.2913 | 7 | *Harpalus sp.* | 0.0000 |
| 6 | *Harpalus rufipes* | 0.3615 | 7 | *Pterostichus sp.* | 0.0000 |
| 6 | *Anchomenus dorsalis* | 0.3896 | 7 | *Anisodactylus signatus* | 0.0000 |
| 6 | *Cylindera germanica* | 0.1368 | 7 | *Agonum muelleri* | 0.3653 |
| 6 | *Amara cf ingenua* | 0.0000 | 7 | *Amara plebeja* | 0.0000 |
| 6 | *Harpalus affinis* | 0.2233 | 7 | *Amara lucida* | 0.0000 |
| 6 | *Amara familiaris* | 0.0227 | 7 | *Anisodactylus binotatus* | 0.2100 |
| 6 | *Clivina fossor* | 0.1512 | 7 | *Amara similata* | 0.1558 |
| 6 | *Harpalus distinguendus* | 0.0000 | 7 | *Amara lunicollis* | 0.0000 |
| 6 | *Carabus granulatus* | 0.1040 | 7 | *Amara sp.* | 0.1129 |
| 6 | acy.pis_210 (*A. pisum*) | 0.5057 | 7 | *Harpalus rufipes* | 0.5926 |
| 6 | Bemb_152 (*Bembidion* genus) | 0.1930 | 7 | *Anchomenus dorsalis* | 0.0000 |
| 6 | btl.thrp_208 (beetles/thrips) | 0.0734 | 7 | *Cylindera germanica* | 0.0000 |
| 6 | earth_85 (earthworms) | 0.6280 | 7 | *Amara cf ingenua* | 0.0000 |
| 6 | Harp_349 (*Harpalus* genus) | 0.0000 | 7 | *Harpalus affinis* | 0.4138 |
| 6 | hover_86 (hoverflies) | 0.3295 | 7 | *Amara familiaris* | 0.0816 |
| 6 | lac.wng_390 (lacewings) | 0.0000 | 7 | *Clivina fossor* | 0.0414 |
| 6 | lady.b_116 (ladybugs) | 0.0437 | 7 | *Harpalus distinguendus* | 0.0000 |
| 6 | met.dir_105 (*M. dirhodum*) | 0.5984 | 7 | *Carabus granulatus* | 0.1704 |
| 6 | oul.mel_248 (*O. melanopus)* | 0.1337 | 7 | acy.pis_210 (*A. pisum*) | 0.2654 |
| 6 | Phi car_245 (*P. carbonarius*) | 0.1687 | 7 | Bemb_152 (*Bembidion* genus) | 0.5106 |

**Supplementary Table 11** – continued.

| Session | Taxa | Eigen. centrality |
| --- | --- | --- |
| 7 | btl.thrp_208 (beetles/thrips) | 0.0863 |
| 7 | earth_85 (earthworms) | 0.7995 |
| 7 | Harp_349 (*Harpalus* genus) | 0.2335 |
| 7 | hover_86 (hoverflies) | 0.3730 |
| 7 | lac.wng_390 (lacewings) | 0.0000 |
| 7 | lady.b_116 (ladybugs) | 0.0935 |
| 7 | met.dir_105 (*M. dirhodum*) | 0.5940 |
| 7 | oul.mel_248 (*O. melanopus)* | 0.3665 |
| 7 | Phi car_245 (*P. carbonarius*) | 0.0863 |
| 7 | Phi cog_216 (*P. cognatus*) | 0.6039 |
| 7 | Poe_112 (*Poecilus* genus) | 0.6418 |
| 7 | Ptero_166 (*Pterostichus* genus) | 0.4191 |
| 7 | rho.pad_136 (*R. padii*) | 0.7510 |
| 7 | sit.ave_304 (*S. avenae*) | 0.0289 |
| 7 | spiders_258 (spiders) | 0.5806 |
| 7 | springt_231 (springtails) | 1.0000 |

**Supplementary Table 12** – Eigenvector centrality for predators and prey (PCR codes) taxa 2021.

| Session | Taxa | Eigen. centrality | Session | Taxa | Eigen. centrality |
| --- | --- | --- | --- | --- | --- |
| 1 | *Anisodactilus signatus* | 0.0000 | 1 | springt_231 (springtails) | 1.0000 |
| 1 | *Agonum muelleri* | 0.0000 | 2 | *Anisodactilus signatus* | 0.0000 |
| 1 | *Carabus granulatus* | 0.0000 | 2 | *Agonum muelleri* | 0.0000 |
| 1 | *Calathus melanocephalus* | 0.0000 | 2 | *Carabus granulatus* | 0.0000 |
| 1 | *Anisodactilus binotatus* | 0.0000 | 2 | *Calathus melanocephalus* | 0.0000 |
| 1 | *Calathus fuscipes* | 0.0000 | 2 | *Anisodactilus binotatus* | 0.0000 |
| 1 | *Agonum sexpunctatum* | 0.0000 | 2 | *Calathus fuscipes* | 0.0000 |
| 1 | *Harpalus affinis* | 0.0964 | 2 | *Agonum sexpunctatum* | 0.0000 |
| 1 | *Amara similata* | 0.0000 | 2 | *Harpalus affinis* | 0.1010 |
| 1 | *Anchomenus dorsalis* | 0.0000 | 2 | *Amara similata* | 0.3114 |
| 1 | Araneae | 0.6321 | 2 | *Anchomenus dorsalis* | 0.1676 |
| 1 | *Bembidion tetracolum* | 0.0000 | 2 | Araneae | 0.4753 |
| 1 | *Bembidion lampros* | 0.0000 | 2 | *Bembidion tetracolum* | 0.0000 |
| 1 | *Clivina fossor* | 0.4181 | 2 | *Bembidion lampros* | 0.4390 |
| 1 | *Harpalus distinguendus* | 0.1493 | 2 | *Clivina fossor* | 0.0000 |
| 1 | *Amara aenea* | 0.1493 | 2 | *Harpalus distinguendus* | 0.0000 |
| 1 | *Cylindera germanica* | 0.0000 | 2 | *Amara aenea* | 0.2805 |
| 1 | *Harpalus rufipes* | 0.0000 | 2 | *Cylindera germanica* | 0.0000 |
| 1 | *Pterostichus melanarius* | 0.0000 | 2 | *Harpalus rufipes* | 0.0000 |
| 1 | *Amara familiaris* | 0.0000 | 2 | *Pterostichus melanarius* | 0.0000 |
| 1 | *Philonthus cognatus* | 0.1493 | 2 | *Amara familiaris* | 0.0000 |
| 1 | *Bembidion properans* | 0.6176 | 2 | *Philonthus cognatus* | 0.9106 |
| 1 | *Poecilus cupreus* | 0.7119 | 2 | *Bembidion properans* | 0.8484 |
| 1 | *Bembidion quadrimaculatum* | 0.1830 | 2 | *Poecilus cupreus* | 0.8604 |
| 1 | *Poecilus versicolor* | 0.5042 | 2 | *Bembidion quadrimaculatum* | 0.0000 |
| 1 | *Loricera pillicornis* | 0.0000 | 2 | *Poecilus versicolor* | 0.4582 |
| 1 | *Tachyporous ruficollis* | 0.0000 | 2 | *Loricera pillicornis* | 0.3681 |
| 1 | *Nebria brevicollis* | 0.1493 | 2 | *Tachyporous ruficollis* | 0.4746 |
| 1 | *Tachyporous rufipes* | 0.0000 | 2 | *Nebria brevicollis* | 0.1268 |
| 1 | *Philonthus carbonarius* | 0.1493 | 2 | *Tachyporous rufipes* | 0.0000 |
| 1 | acy.pis_210 (*A. pisum*) | 0.0944 | 2 | *Philonthus carbonarius* | 0.0000 |
| 1 | Bemb_152 (*Bembidion* genus) | 0.3823 | 2 | acy.pis_210 (*A. pisum*) | 0.3504 |
| 1 | btl.thrp_208 (beetles/thrips) | 0.1866 | 2 | Bemb_152 (*Bembidion* genus) | 0.3579 |
| 1 | earth_85 (earthworms) | 0.6457 | 2 | btl.thrp_208 (beetles/thrips) | 0.0000 |
| 1 | Harp_349 (*Harpalus* genus) | 0.0753 | 2 | earth_85 (earthworms) | 0.6584 |
| 1 | hover_86 (hoverflies) | 0.1063 | 2 | Harp_349 (*Harpalus* genus) | 0.0000 |
| 1 | lac.wng_390 (lacewings) | 0.0000 | 2 | hover_86 (hoverflies) | 0.0000 |
| 1 | lady.b_116 (ladybugs) | 0.0000 | 2 | lac.wng_390 (lacewings) | 0.0000 |
| 1 | met.dir_105 (*M. dirhodum*) | 0.1546 | 2 | lady.b_116 (ladybugs) | 0.0000 |
| 1 | oul.mel_248 (*O. melanopus)* | 0.2788 | 2 | met.dir_105 (*M. dirhodum*) | 0.5969 |
| 1 | Phi car_245 (*P. carbonarius*) | 0.0000 | 2 | oul.mel_248 (*O. melanopus)* | 0.4423 |
| 1 | Phi cog_216 (*P. cognatus*) | 0.1063 | 2 | Phi car_245 (*P. carbonarius*) | 0.0000 |
| 1 | Poe_112 (*Poecilus* genus) | 0.0922 | 2 | Phi cog_216 (*P. cognatus*) | 0.5025 |
| 1 | Ptero_166 (*Pterostichus* genus) | 0.0000 | 2 | Poe_112 (*Poecilus* genus) | 0.5701 |
| 1 | rho.pad_136 (*R. padii*) | 0.2738 | 2 | Ptero_166 (*Pterostichus* genus) | 0.0000 |
| 1 | sit.ave_304 (*S. avenae*) | 0.0000 | 2 | rho.pad_136 (*R. padii*) | 0.3016 |
| 1 | spiders_258 (spiders) | 0.2258 | 2 | sit.ave_304 (*S. avenae*) | 0.0480 |

**Supplementary Table 12** – continued.

| Session | Taxa | Eigen. centrality | Session | Taxa | Eigen. centrality |
| --- | --- | --- | --- | --- | --- |
| 2 | spiders_258 (spiders) | 0.7757 | 3 | sit.ave_304 (*S. avenae*) | 0.0983 |
| 2 | springt_231 (springtails) | 1.0000 | 3 | spiders_258 (spiders) | 0.4589 |
| 3 | *Anisodactilus signatus* | 0.0000 | 3 | springt_231 (springtails) | 0.9505 |
| 3 | *Agonum muelleri* | 0.2236 | 4 | *Anisodactilus signatus* | 0.0447 |
| 3 | *Carabus granulatus* | 0.0000 | 4 | *Agonum muelleri* | 0.1338 |
| 3 | *Calathus melanocephalus* | 0.0483 | 4 | *Carabus granulatus* | 0.0000 |
| 3 | *Anisodactilus binotatus* | 0.0000 | 4 | *Calathus melanocephalus* | 0.0000 |
| 3 | *Calathus fuscipes* | 0.0000 | 4 | *Anisodactilus binotatus* | 0.4625 |
| 3 | *Agonum sexpunctatum* | 0.2085 | 4 | *Calathus fuscipes* | 0.1307 |
| 3 | *Harpalus affinis* | 0.3472 | 4 | *Agonum sexpunctatum* | 0.0620 |
| 3 | *Amara similata* | 0.7085 | 4 | *Harpalus affinis* | 0.4950 |
| 3 | *Anchomenus dorsalis* | 0.0000 | 4 | *Amara similata* | 0.5812 |
| 3 | Araneae | 0.5685 | 4 | *Anchomenus dorsalis* | 0.0599 |
| 3 | *Bembidion tetracolum* | 0.0000 | 4 | Araneae | 0.4367 |
| 3 | *Bembidion lampros* | 0.2794 | 4 | *Bembidion tetracolum* | 0.1002 |
| 3 | *Clivina fossor* | 0.0403 | 4 | *Bembidion lampros* | 0.4307 |
| 3 | *Harpalus distinguendus* | 0.0000 | 4 | *Clivina fossor* | 0.2789 |
| 3 | *Amara aenea* | 0.8714 | 4 | *Harpalus distinguendus* | 0.0000 |
| 3 | *Cylindera germanica* | 0.0000 | 4 | *Amara aenea* | 0.6495 |
| 3 | *Harpalus rufipes* | 0.1787 | 4 | *Cylindera germanica* | 0.0000 |
| 3 | *Pterostichus melanarius* | 0.7744 | 4 | *Harpalus rufipes* | 0.3605 |
| 3 | *Amara familiaris* | 0.0000 | 4 | *Pterostichus melanarius* | 0.7040 |
| 3 | *Philonthus cognatus* | 1.0000 | 4 | *Amara familiaris* | 0.0000 |
| 3 | *Bembidion properans* | 0.8709 | 4 | *Philonthus cognatus* | 0.6880 |
| 3 | *Poecilus cupreus* | 0.9522 | 4 | *Bembidion properans* | 0.7196 |
| 3 | *Bembidion quadrimaculatum* | 0.0000 | 4 | *Poecilus cupreus* | 0.6738 |
| 3 | *Poecilus versicolor* | 0.3651 | 4 | *Bembidion quadrimaculatum* | 0.1002 |
| 3 | *Loricera pillicornis* | 0.1618 | 4 | *Poecilus versicolor* | 0.4230 |
| 3 | *Tachyporous ruficollis* | 0.2010 | 4 | *Loricera pillicornis* | 0.1018 |
| 3 | *Nebria brevicollis* | 0.7628 | 4 | *Tachyporous ruficollis* | 0.3001 |
| 3 | *Tachyporous rufipes* | 0.0000 | 4 | *Nebria brevicollis* | 0.5554 |
| 3 | *Philonthus carbonarius* | 0.2550 | 4 | *Tachyporous rufipes* | 0.0419 |
| 3 | acy.pis_210 (*A. pisum*) | 0.5902 | 4 | *Philonthus carbonarius* | 0.6141 |
| 3 | Bemb_152 (*Bembidion* genus) | 0.4028 | 4 | acy.pis_210 (*A. pisum*) | 0.5086 |
| 3 | btl.thrp_208 (beetles/thrips) | 0.0388 | 4 | Bemb_152 (*Bembidion* genus) | 0.3425 |
| 3 | earth_85 (earthworms) | 0.8696 | 4 | btl.thrp_208 (beetles/thrips) | 0.0262 |
| 3 | Harp_349 (*Harpalus* genus) | 0.0683 | 4 | earth_85 (earthworms) | 0.5411 |
| 3 | hover_86 (hoverflies) | 0.4831 | 4 | Harp_349 (*Harpalus* genus) | 0.0000 |
| 3 | lac.wng_390 (lacewings) | 0.0000 | 4 | hover_86 (hoverflies) | 0.3796 |
| 3 | lady.b_116 (ladybugs) | 0.0595 | 4 | lac.wng_390 (lacewings) | 0.0000 |
| 3 | met.dir_105 (*M. dirhodum*) | 0.9966 | 4 | lady.b_116 (ladybugs) | 0.0000 |
| 3 | oul.mel_248 (*O. melanopus)* | 0.8575 | 4 | met.dir_105 (*M. dirhodum*) | 0.9723 |
| 3 | Phi car_245 (*P. carbonarius*) | 0.0000 | 4 | oul.mel_248 (*O. melanopus)* | 0.6995 |
| 3 | Phi cog_216 (*P. cognatus*) | 0.6692 | 4 | Phi car_245 (*P. carbonarius*) | 0.1215 |
| 3 | Poe_112 (*Poecilus* genus) | 0.7035 | 4 | Phi cog_216 (*P. cognatus*) | 0.6989 |
| 3 | Ptero_166 (*Pterostichus* genus) | 0.4690 | 4 | Poe_112 (*Poecilus* genus) | 0.3344 |
| 3 | rho.pad_136 (*R. padii*) | 0.7066 | 4 | Ptero_166 (*Pterostichus* genus) | 0.1981 |

**Supplementary Table 12** – continued.

| Session | Taxa | Eigen. centrality | Session | Taxa | Eigen. centrality |
| --- | --- | --- | --- | --- | --- |
| 4 | rho.pad_136 (*R. padii*) | 0.7459 | 5 | Ptero_166 (*Pterostichus* genus) | 0.4795 |
| 4 | sit.ave_304 (*S. avenae*) | 0.0493 | 5 | rho.pad_136 (*R. padii*) | 0.2476 |
| 4 | spiders_258 (spiders) | 0.5597 | 5 | sit.ave_304 (*S. avenae*) | 0.1767 |
| 4 | springt_231 (springtails) | 1.0000 | 5 | spiders_258 (spiders) | 0.5791 |
| 5 | *Anisodactilus signatus* | 0.0000 | 5 | springt_231 (springtails) | 1.0000 |
| 5 | *Agonum muelleri* | 0.0811 | 6 | *Anisodactilus signatus* | 0.3046 |
| 5 | *Carabus granulatus* | 0.1797 | 6 | *Agonum muelleri* | 0.1552 |
| 5 | *Calathus melanocephalus* | 0.0000 | 6 | *Carabus granulatus* | 0.0000 |
| 5 | *Anisodactilus binotatus* | 0.5227 | 6 | *Calathus melanocephalus* | 0.0000 |
| 5 | *Calathus fuscipes* | 0.1308 | 6 | *Anisodactilus binotatus* | 0.3686 |
| 5 | *Agonum sexpunctatum* | 0.2850 | 6 | *Calathus fuscipes* | 0.1027 |
| 5 | *Harpalus affinis* | 0.1642 | 6 | *Agonum sexpunctatum* | 0.2326 |
| 5 | *Amara similata* | 0.6567 | 6 | *Harpalus affinis* | 0.2040 |
| 5 | *Anchomenus dorsalis* | 0.1765 | 6 | *Amara similata* | 0.5175 |
| 5 | Araneae | 0.3419 | 6 | *Anchomenus dorsalis* | 0.3205 |
| 5 | *Bembidion tetracolum* | 0.0000 | 6 | Araneae | 0.4150 |
| 5 | *Bembidion lampros* | 0.0696 | 6 | *Bembidion tetracolum* | 0.2031 |
| 5 | *Clivina fossor* | 0.2800 | 6 | *Bembidion lampros* | 0.2593 |
| 5 | *Harpalus distinguendus* | 0.0000 | 6 | *Clivina fossor* | 0.0241 |
| 5 | *Amara aenea* | 0.5219 | 6 | *Harpalus distinguendus* | 0.0000 |
| 5 | *Cylindera germanica* | 0.1308 | 6 | *Amara aenea* | 0.2089 |
| 5 | *Harpalus rufipes* | 0.2499 | 6 | *Cylindera germanica* | 0.0778 |
| 5 | *Pterostichus melanarius* | 0.8323 | 6 | *Harpalus rufipes* | 0.5837 |
| 5 | *Amara familiaris* | 0.0436 | 6 | *Pterostichus melanarius* | 0.8454 |
| 5 | *Philonthus cognatus* | 0.8566 | 6 | *Amara familiaris* | 0.0000 |
| 5 | *Bembidion properans* | 0.6052 | 6 | *Philonthus cognatus* | 0.6921 |
| 5 | *Poecilus cupreus* | 0.8419 | 6 | *Bembidion properans* | 0.2936 |
| 5 | *Bembidion quadrimaculatum* | 0.2441 | 6 | *Poecilus cupreus* | 0.5959 |
| 5 | *Poecilus versicolor* | 0.3835 | 6 | *Bembidion quadrimaculatum* | 0.0000 |
| 5 | *Loricera pillicornis* | 0.4594 | 6 | *Poecilus versicolor* | 0.2092 |
| 5 | *Tachyporous ruficollis* | 0.1525 | 6 | *Loricera pillicornis* | 0.2117 |
| 5 | *Nebria brevicollis* | 0.0000 | 6 | *Tachyporous ruficollis* | 0.1735 |
| 5 | *Tachyporous rufipes* | 0.0000 | 6 | *Nebria brevicollis* | 0.0783 |
| 5 | *Philonthus carbonarius* | 0.5891 | 6 | *Tachyporous rufipes* | 0.0000 |
| 5 | acy.pis_210 (*A. pisum*) | 0.6342 | 6 | *Philonthus carbonarius* | 0.2618 |
| 5 | Bemb_152 (*Bembidion* genus) | 0.4424 | 6 | acy.pis_210 (*A. pisum*) | 0.3094 |
| 5 | btl.thrp_208 (beetles/thrips) | 0.0476 | 6 | Bemb_152 (*Bembidion* genus) | 0.2555 |
| 5 | earth_85 (earthworms) | 0.4216 | 6 | btl.thrp_208 (beetles/thrips) | 0.0646 |
| 5 | Harp_349 (*Harpalus* genus) | 0.0000 | 6 | earth_85 (earthworms) | 0.6848 |
| 5 | hover_86 (hoverflies) | 0.5862 | 6 | Harp_349 (*Harpalus* genus) | 0.0061 |
| 5 | lac.wng_390 (lacewings) | 0.0000 | 6 | hover_86 (hoverflies) | 0.4007 |
| 5 | lady.b_116 (ladybugs) | 0.0125 | 6 | lac.wng_390 (lacewings) | 0.0000 |
| 5 | met.dir_105 (*M. dirhodum*) | 0.6783 | 6 | lady.b_116 (ladybugs) | 0.0403 |
| 5 | oul.mel_248 (*O. melanopus)* | 0.5143 | 6 | met.dir_105 (*M. dirhodum*) | 0.3800 |
| 5 | Phi car_245 (*P. carbonarius*) | 0.2757 | 6 | oul.mel_248 (*O. melanopus)* | 0.3383 |
| 5 | Phi cog_216 (*P. cognatus*) | 0.8780 | 6 | Phi car_245 (*P. carbonarius*) | 0.1304 |
| 5 | Poe_112 (*Poecilus* genus) | 0.6264 | 6 | Phi cog_216 (*P. cognatus*) | 0.3436 |

**Supplementary Table 12** – continued.

| Session | Taxa | Eigen. centrality |
| --- | --- | --- |
| 6 | Poe_112 (*Poecilus* genus) | 0.4252 |
| 6 | Ptero_166 (*Pterostichus* genus) | 0.3194 |
| 6 | rho.pad_136 (*R. padii*) | 0.6089 |
| 6 | sit.ave_304 (*S. avenae*) | 0.0000 |
| 6 | spiders_258 (spiders) | 0.6213 |
| 6 | springt_231 (springtails) | 1.0000 |


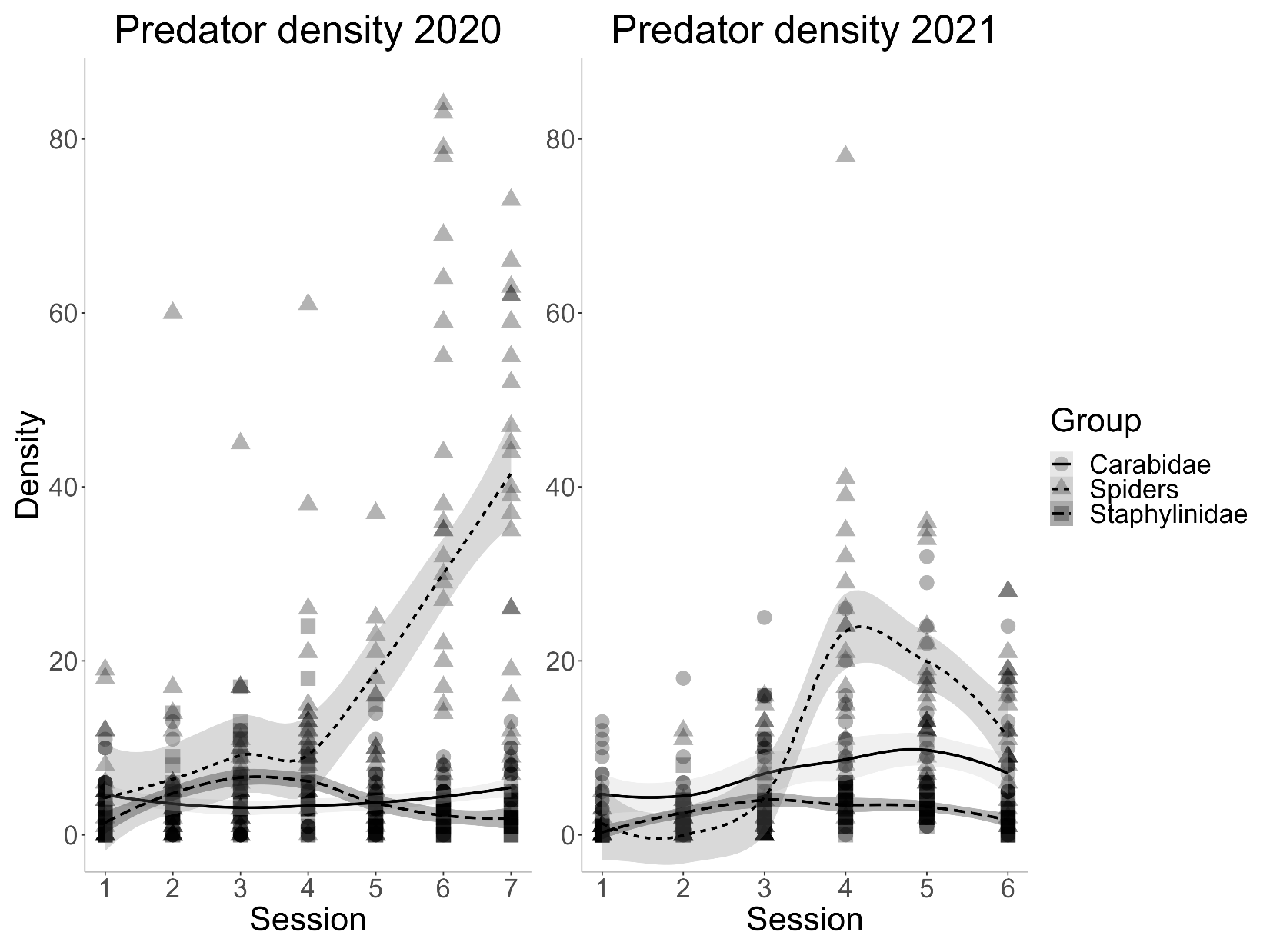


**Supplementary Figure 15** – Predator group density across sampling seasons in 2020 and 2021.
